# Efficacy of Various Hypoglycemic Agents in the Treatment of Patients With Nonalcoholic Liver Disease With or Without Diabetes： A Network Meta-Analysis

**DOI:** 10.3389/fendo.2021.649018

**Published:** 2021-03-24

**Authors:** Jingxuan Lian, Jianfang Fu

**Affiliations:** Department of Endocrinology, Xijing Hospital of Air Force Medical University, Xi’an, China

**Keywords:** NAFLD (nonalcoholic fatty liver disease), Diabetes Mellitus Type 2, anti-diabetic drugs, network meta analyses, efficacy

## Abstract

**Objective:**

To comprehensively evaluate and compare the therapeutic effects of various hypoglycemic agents in NAFLD patients with or without diabetes.

**Methods:**

All literature from the Cochrane Central Register of Controlled Trials (CENTRAL), PubMed, and Clinical Trials was searched, and the language was limited to English. Two reviewers independently assessed study eligibility, continuous data extraction, and independent assessment of bias risk. Our primary outcomes were alanine aminotransferase (ALT), aspartate aminotransferase (AST) and triglyceride levels, while our secondary outcomes were high-density lipoprotein (HDL) and low-density lipoprotein (LDL) levels, body weight, BMI, and fasting glucose and glycosylated hemoglobin (HbA1c) levels.

**Results:**

The review identified 20 eligible trials that met the inclusion criteria. We found that, compared to other drugs, thiazolidinediones, especially pioglitazone, had a greater effect on the levels of ALT (-8.01 (95% CI -14.3 to 2.02)) and AST (-5.0 (95% CI -9.21 to -1,22)) and other biological indicators, but they were also associated with an increased risk of weight gain (3.62 (95% CI 2.25 to 4.99) and increased BMI (0.59 (95% Cl -0.13 to 1.29). GLP1 RAs and metformin also had better therapeutic effects than other drugs as measured by the levels of ALT (liraglutide: -9.36 (95% Cl -18 to -0.34), metformin: -2.84 (95% CI -11.09 to 5.28)) and AST (liraglutide: -5.14 (95% CI -10.69 to 0.37), metformin: -2.39 (95% CI -7.55, 2.49)) and other biological indicators.

**Conclusion:**

Despite the significant risk of weight gain, thiazolidinediones, especially pioglitazone, are beneficial in normalizing liver and glucose metabolism in NAFLD patients. In clinical practice, we believe that GLP1 RAs such as liraglutide and exenatide or metformin can be used in combination to offset the risk of weight gain associated with thiazolidinediones. However, long-term studies are still needed to verify the efficacy and safety of individual hypoglycemic agents.

**Systematic Review Registration:**

[PROSPERO], identifier [CRD42020212025].

Nonalcoholic fatty liver disease (NAFLD) is one of the most common liver diseases worldwide in both adults and children. Studies have shown that the prevalence of NAFLD is approximately 25% in adults and 8% in adolescents (1, 2). In addition, with the global prevalence of obesity, type 2 diabetes (T2DM) and sedentary lifestyles, NAFLD prevalence is expected to increase exponentially in the next few decades. NAFLD is a clinical syndrome that includes a variety of related diseases and liver complications, including hepatocellular carcinoma (HCC) (3). NAFLD is also considered to be the main indication for future liver transplantation (4).

The individual role of nonalcoholic fatty liver disease should not be underestimated; in most cases, but not in specific genetically determined hepatic steatosis, the increasing prevalence of NAFLD is associated with the increasing prevalence of other noncommunicable diseases including insulin resistance, type 2 diabetes, CVD, type 2 diabetes-associated cancer, and advanced liver diseases such as hepatic cirrhosis and hepatic cancer. In addition, NAFLD is not often found in individuals who are obese but metabolically healthy (5, 6). Studies have shown that the all-cause mortality of individuals with NAFLD is higher than that of the general population (7), making it an important health problem. Despite the heavy burden imposed by NAFLD, there is still no effective treatment strategy.

NAFLD is currently treated clinically with a combination of drugs. Although no drugs have been specifically approved for the treatment of NAFLD, many hypoglycemic agents have been tested in NAFLD patients. In addition, many new compounds for the treatment of type 2 diabetes are being developed, and their efficacy in treating NAFLD has also been tested in clinical trials (8). In patients with NAFLD, a combination of lifestyle changes and treatment with hypoglycemic agents has been shown to reduce liver fat accumulation and to have other effects in slowing down the process of NASH and fibrosis.

Although there is much medical evidence regarding the treatment of NAFLD with hypoglycemic agents, only a very limited number of studies and reports have compared the therapeutic effects of different hypoglycemic agents in NAFLD patients. Therefore, it is difficult to draw conclusions about the efficacy of different types of hypoglycemic agents in treating NAFLD.

The purpose of this study was to comprehensively evaluate the therapeutic effects of various hypoglycemic agents that have been approved for the treatment of NAFLD patients with or without diabetes through systematic reviews and network meta-analysis (NMA).

## Methods

The systematic review scheme used in this study is reported in accordance with the preferred reporting items listed in the guidelines for systematic review and meta-analysis (PRISMA-P) (9). Our PROSPERO registration ID is CRD42020212025.

### Data Sources and Search Strategy

We performed an electronic search of the following databases: Cochrane Central Register of Controlled Trials (CENTRAL), PubMed and ClinicalTrials. We searched each database for articles published before September 2020 in English (the complete search strategy is shown in **Appendix 1** of the **Supplementary Materials**). In all phases of the research, we followed the preferred reporting items for systematic reviews and network meta-analysis guidelines.

### Inclusion and Exclusion Criteria

We included all randomized controlled trials of the efficacy of hypoglycemic agents in NAFLD patients between 18 and 70 years of age with and without diabetes. Studies with an intervention time <4 weeks or that included patients with any non-NAFLD chronic liver disease related to any disease other than diabetes and studies that did not distinguish patients in the subgroup analysis were excluded. In addition, prescription drugs and food supplements and other nonhypoglycemic agents such as combinations of liver-protective drugs were excluded. For the crossover RCTS, the carrying effect is considered; therefore, we used the data from the first phase of the study.

### Study Selection and Data Extraction

Two reviewers independently screened and confirmed the research and resolved their differences through discussion. In addition, manual searches of references included in published systematic reviews and meta-analyses were performed to ensure that no relevant studies were missed. According to the previously defined standard, the data were independently extracted into an Excel spreadsheet. For each included study, we extracted data such as study duration, trial design, intervention measures and their duration, demographic characteristics and baseline characteristics.

Our primary outcomes were alanine aminotransferase (ALT), aspartate aminotransferase (AST) and triglyceride levels, and our secondary outcomes were high-density lipoprotein (HDL) and low-density lipoprotein (LDL) levels, weight, BMI and fasting plasma glucose and glycosylated hemoglobin (HbA1c) levels.

### Quality Assessment

The Cochrane collaborative risk assessment tool was used to assess the risk of bias for each included study. The following aspects were evaluated: random sequence generation, allocation hiding, blindness of participants and personnel, blindness of result evaluation, incomplete result data, selective reporting and other biases.

### Data Synthesis and Statistical Analysis

A random effects network meta-analysis was conducted within the Bayesian framework to assess the relative effectiveness of each hypoglycemic agent. Bayesian network meta-analysis is a generalization of traditional meta-analysis that allows all evidence (direct and indirect) to be considered simultaneously. It can be applied to any connected evidence network. The posterior densities for all unknown parameters were estimated using the MCMC (Markov chain Monte Carlo) method for each model. Each chain used 50,000 iterations with a burn-in of 20,000.

The league table was used to show the specific therapeutic effects of different hypoglycemic agents after comparison. Using the surface under the cumulative sorting curve (SUCRA) to estimate the sorting probability of each treatment, the treatment hierarchy was obtained (10). SUCRA is a percentage that is interpreted as the percentage at which the curative effect of a treatment ranks first without any uncertainty. When a treatment is definitely the best, it equals 1, and when the treatment is definitely the worst, it equals 0. For each result, both fixed effects (FE) and random effects (RE) models were run, and more appropriate models based on deviance information criteria (DIC), average posterior residual deviance and I^2^ were also used. All outcomes were analyzed using the consistency model and the inconsistency model, and the overall heterogeneity was compared based on the differences in DIC and I^2^. The NMA results are presented as the mean treatment differences and the associated 95% confidence intervals (CI).

NMAs combine all available evidence from clinical trials to estimate treatment effectiveness. Since this involves a combination of direct and indirect measures of effect, it is important to examine whether these two sources of evidence are consistent with each other. Therefore, all NMAs evaluated inconsistencies using R software. If there was evidence of substantial inconsistencies, the specific reasons that led to the inconsistency of the results were determined by reviewing the corresponding studies in a further analysis. Stata 13 software was used to draw the network evidence graph, and R software (3.4.1) and the GEMTC software package were used to conduct the network meta-analysis.

## Results

### Identified Publications

**Figure 1:**
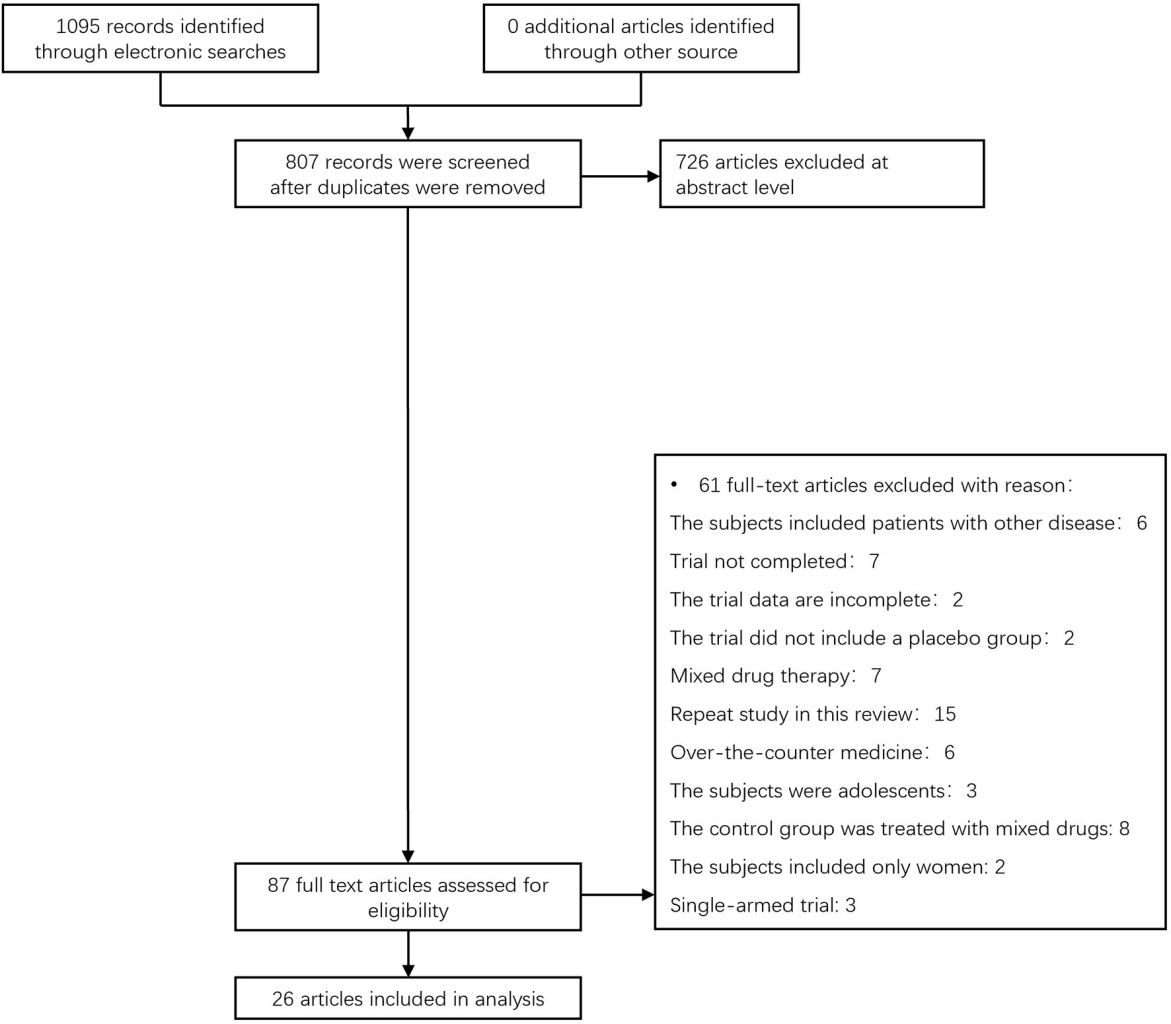
The process for inclusion of RCTs involving patients with nonalcoholic liver disease with or without diabetes, RCTs, and randomized clinical trials.

We found a total of 1095 articles from the target database, among which 87 full-text papers fulfilled the study criteria, and 61 full-text papers were excluded according to the research exclusion criteria. A total of 26 RCTS of hypoglycemic agents in the treatment of non-alcoholic liver disease patients with or without diabetes met the inclusion criteria (11–36) (**Figure 1**). Most of the trials were conducted in the United States(6 trials), China(7 trials) and Iran(4 trials), there are also some trails in Japan, the Netherlands, Switzerland and other countries. All the trial interventions lasted more than 2 months, with a maximum duration of 96 weeks (**Supplementary Materials** **Table S1**).

**Figure 2:**
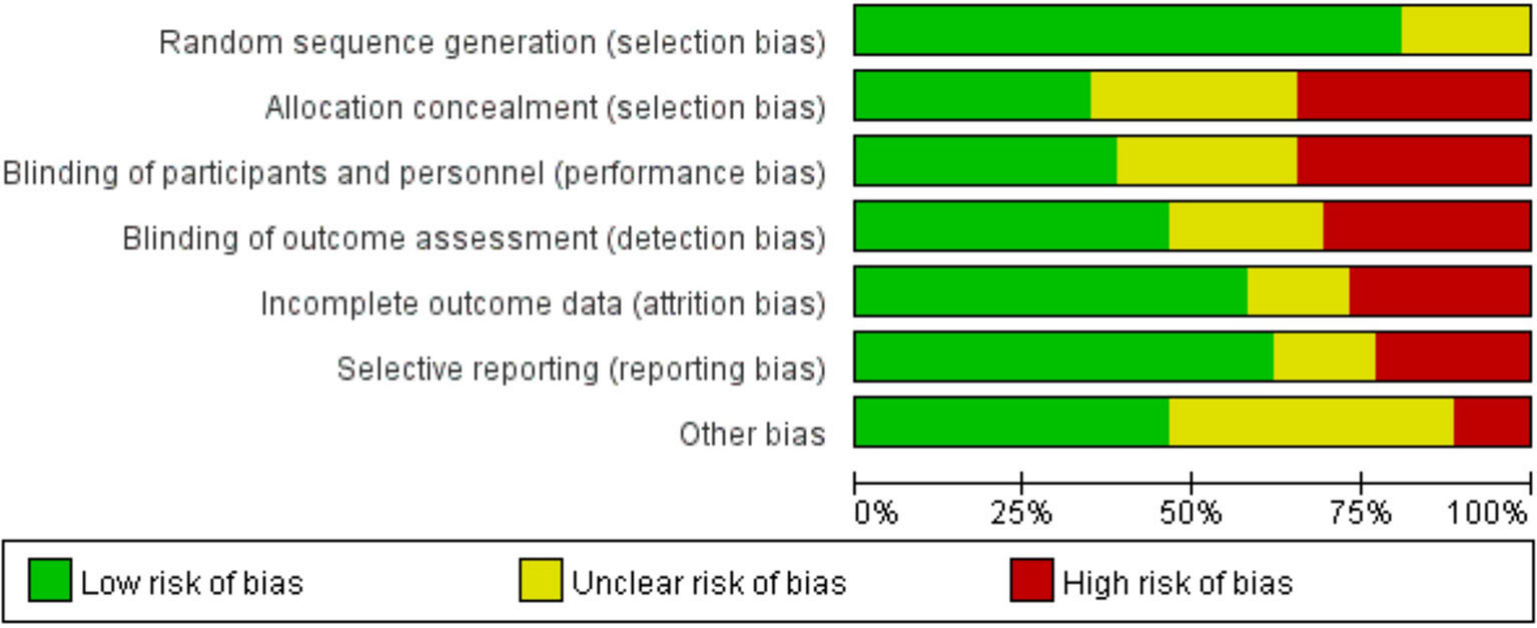
Risk of bias graph showing the review authors’ judgments about each risk-of-bias item presented as percentages across all included studies.

Through risk assessment using the Cochrane Collaboration risk assessment tool, it was found that the overall risk was moderate (**Figures 2** **,** **3**); risk was mainly caused by allocation concealment, selective reporting, incomplete data, and unclear blind reporting in some studies.

**Figure 3:**
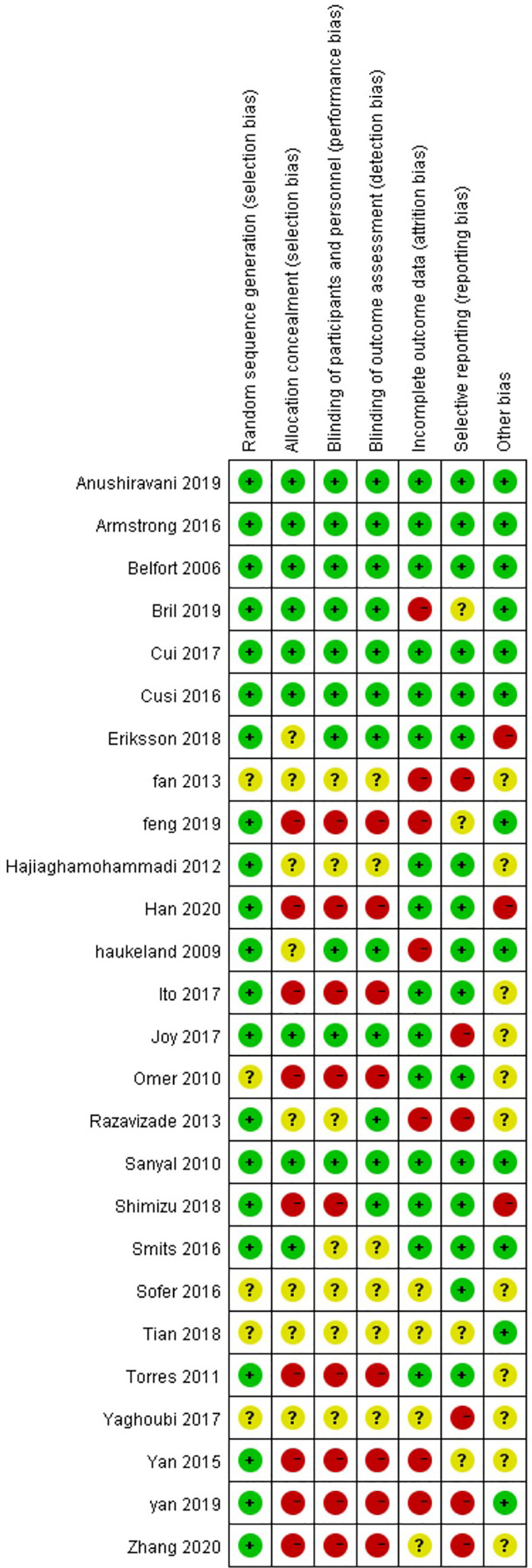
Risk of bias summary for the review authors’ judgments about each risk-of-bias item for each included study.

**Figure 4:**
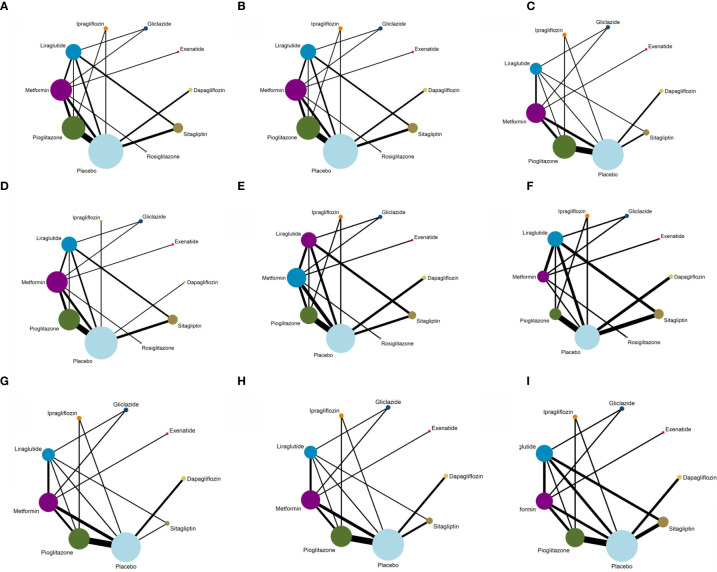
Evidence structure of different outcome indicators used in the network meta-analysis. **(A)** Network plot for ALT. **(B)** Network plot for AST. **(C)** Network plot for Triglyceride. **(D)** Network plot for BMI. **(E)** Network plot for FPG. **(F)** Network plot for HbA1c. **(G)** Network plot for HDL. **(H)** Network plot for LDL. **(I)** Network plot for Weight.

All the trials were included in the research network, and it was found through the network evidence graph (**Figure 4**) that the controlled clinical trials of the treatment of NAFLD patients with glucose-lowering drugs mainly included trials of metformin, pioglitazone, liraglutide, and sitagliptin and that there were few studies on other glucose-lowering drugs. All drugs had primary and secondary outcomes except rosiglitazone; for that drug, secondary analysis results could not be obtained due to the small number of relevant trials.

### Network Meta-Analysis Results

#### ALT

**Figure 5:**
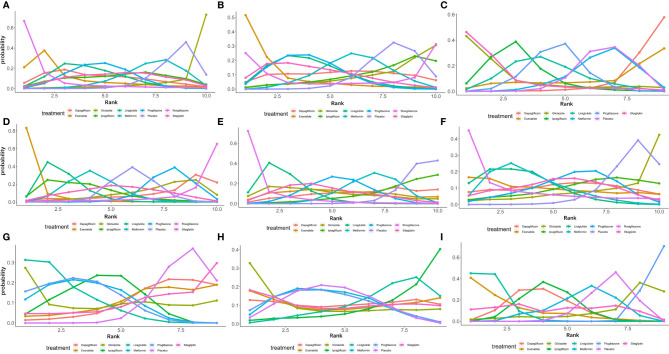
Ranking probabilities of different hypoglycemic agents for different outcome indicators. **(A)** Ranking probability of different hypoglycemic agents on ALT. **(B)** Ranking probability of different hypoglycemic agents on AST. **(C)** Ranking probability of different hypoglycemic agents on Triglyceride. **(D)** Ranking probability of different hypoglycemic agents on BMI. **(E)** Ranking probability of different hypoglycemic agents on FPG. **(F)** Ranking probability of different hypoglycemic agents on HbA1c. **(G)** Ranking probability of different hypoglycemic agents on HDL. **(H)** Ranking probability of different hypoglycemic agents on LDL. **(I)** Ranking probability of different hypoglycemic agents on Weight.

**Figure 6:**
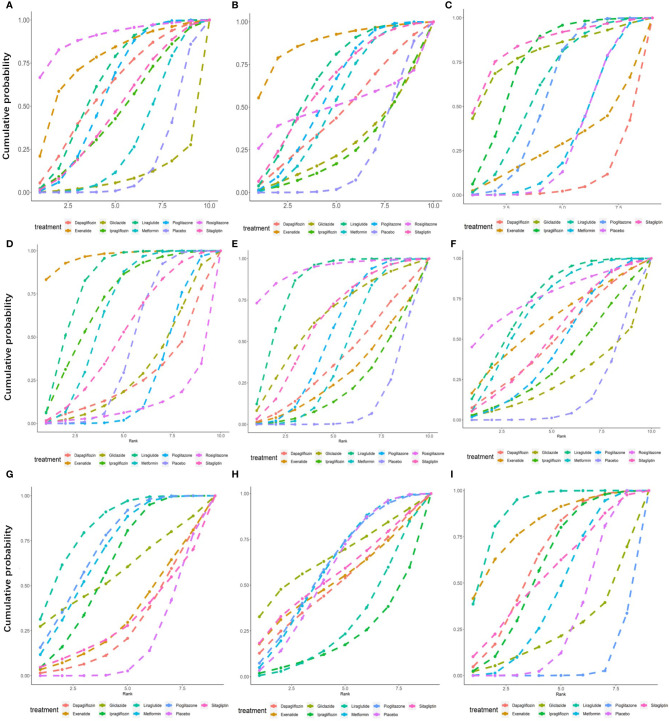
Surface under the cumulative sorting curve (SUCRA) of different hypoglycemic agents for different outcome indicators. **(A)** Cumulative probability of different hypoglycemic agents on ALT. **(B)** Cumulative probability of different hypoglycemic agents on AST. **(C)** Cumulative probability of different hypoglycemic agents on Triglyceride. **(D)** Cumulative probability of different hypoglycemic agents on BMI. **(E)** Cumulative probability of different hypoglycemic agents on FPG. **(F)** Cumulative probability of different hypoglycemic agents on HbA1c. **(G)** Cumulative probability of different hypoglycemic agents on HDL. **(H)** Cumulative probability of different hypoglycemic agents on LDL. **(I)** Cumulative probability of different hypoglycemic agents on Weight.

**Figure 7:**
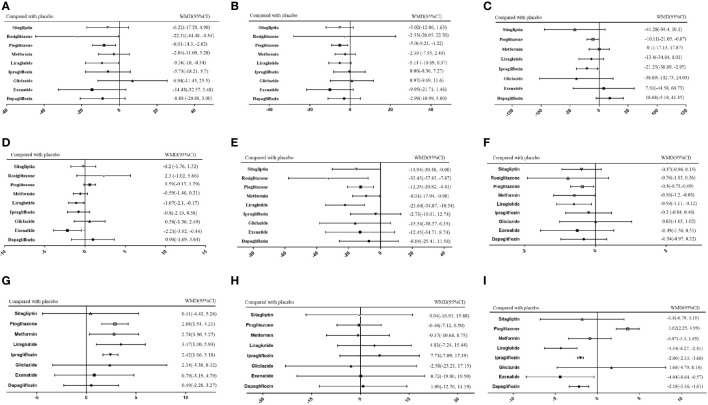
Network meta-analysis of the effects of various hypoglycemic agents on specific outcome indicators. **(A)** Network analysis results of comparison between different hypoglycemic agents on ALT. **(B)** Network analysis results of comparison between different hypoglycemic agents on AST. **(C)** Network analysis results of comparison between different hypoglycemic agents on Triglyceride. **(D)** Network analysis results of comparison between different hypoglycemic agents on BMI. **(E)** Network analysis results of comparison between different hypoglycemic agents on FPG. **(F)** Network analysis results of comparison between different hypoglycemic agents on HbA1c. **(G)** Network analysis results of comparison between different hypoglycemic agents on HDL. **(H)** Network analysis results of comparison between different hypoglycemic agents on LDL. **(I)** Network analysis results of comparison between different hypoglycemic agents on Weight.

As shown in **Figure 5A**, of the drugs for which studies have been reported, exenatide may be most effective at lowering ALT levels in NAFLD patients with or without T2DM, followed by rosiglitazone. Liraglutide and pioglitazone also had good therapeutic effects, while the effects of gliclazide and ipragliflozin were poor. The SUCRA chart shows that the probabilities of exenatide, rosiglitazone, and pioglitazone being among the top three most effective drugs were 85%, 44% and 45%, respectively; the treatment effect ranked placebo, gliclazide and ipragliflozin as the three least effective treatments with probabilities of 68%, 65% and 59%, respectively (**Figure 6A**). A network meta-analysis was shown in **Figure 7A**. The specific results of the comparison of the effects of different drugs on ALT levels are shown in the **Supplementary Materials** (**Table S2**).

#### AST

As shown in **Figure 5B**, rosiglitazone was most likely to be the most effective of all drugs at reducing AST levels in patients with NAFLD with or without type 2 diabetes, followed by exenatide, while gliclazide was less effective even than the placebo. The SUCRA shows that the probability that rosiglitazone is among the top three most effective drugs is 88%. The probability that exenatide is among the top three is 70%, while gliclazide has a probability of 88% of being among the three least effective drugs (**Figure 6B**). The network meta-analysis demonstrated that, compared with the placebo, rosiglitazone was a better choice for improving the AST levels of patients with NAFLD with or without type 2 diabetes, followed by exenatide; gliclazide had the least effect and was significantly less effective than the placebo (**Figure 7B**). The specific results of the comparison of the effects of different drugs on NAFLD patients’ AST levels are shown in the **Supplementary Materials** (**Table S3**).

#### Triglycerides

Gliclazide and sitagliptin were more effective in reducing triglyceride levels than other drugs (**Figure 5C**), and the SUCRA showed that their therapeutic effects ranked in the top three with probabilities of 77% and 84%, respectively. Dapagliflozin and exenatide had probabilities of 95% and 63%, respectively, of being the least effective drugs (**Figure 6C**). A network meta-analysis was shown in **Figure 7C**. The specific results of the comparison between the effects of different drugs on triglyceride levels are shown in the **Supplementary Materials** (**Table S4**).

#### BMI

As shown in **Figure 5D**, in terms of BMI, for NAFLD patients with or without type 2 diabetes, exenatide has obvious advantages over other drugs, and the SUCRA shows that the possibility of its ranking in the top three is as high as 96% (**Figure 6D**). However, thiazolidinedione hypoglycemic agents such as rosiglitazone and pioglitazone carry a significantly higher risk of weight gain than other drugs, and their therapeutic effects rank among the lowest three with 87% and 63% probability, respectively (**Figure 6D**). A network meta-analysis showed that compared with placebo, exenatide, liraglutide, ipragliflozin and metformin led to significant reduction in the weight of NAFLD patients with or without type 2 diabetes (**Figure 7D**). The specific results of the comparison of the effects of different drugs on BMI are shown in the **Supplementary Materials** (**Table S5**).

#### FPG and HbA1c

As shown in **Figure 5E**, rosiglitazone has obvious advantages over other hypoglycemic agents in its ability to reduce fasting blood glucose levels in patients with NAFLD with or without type 2 diabetes. The SUCRA showed that the probability of its being among the top three most effective drugs was 90%; metformin and rosiglitazone also had good efficacy (**Figure 6E**). The network meta-analysis demonstrated that all drugs that act as hypoglycemic agents had a significant therapeutic effect compared with placebo (**Figure 7E**). **Figure 5F** shows that rosiglitazone also has significant advantages over other hypoglycemic agents in improving HbA1c levels in patients with NAFLD with or without type 2 diabetes. In addition, liraglutide and metformin have good therapeutic effects. The SUCRA showed that the probabilities of rosiglitazone, liraglutide and metformin pioglitazone being among the top three most effective drugs at decreasing FPG levels were 66%, 56% and 50%, respectively (**Figure 6F**). Compared with placebo, the network meta-analysis of NAFLD patients with or without type 2 diabetes showed that all of the drugs except gliclazide significantly improved the blood glucose levels of patients (**Figure 7F**). The specific results of the comparison of the effects of different drugs on FPG and HbAc levels are shown in the **Supplementary Material** (**Tables S6**, **S7**).

#### HDL

In terms of HDL, a network meta-analysis showed that liraglutide, metformin, pioglitazone and ipragliflozin can significantly improve the HDL levels of NAFLD patients with or without type 2 diabetes (**Figure 7G**). SUCRA showed that the probabilities that these drugs are among the top three most effective at improving HDL levels were 79%, 52%, 57% and 34%, respectively. The least effective therapies are placebo, dapagliflozin and sitagliptin. The probabilities that these are among the three least effective treatments are 86%, 62% and 59%, respectively (**Figure 6G**). The Ranking probability of different hypoglycemic agents was shown in **Figure 5G**. There are no relevant experimental data for rosiglitazone because it is impossible to analyze its therapeutic effect on HDL due to the lack of relevant RCTs. The specific results of the comparison of the effects of different drugs on HDL levels are shown in the **Supplementary Materials** (**Table S8**).

#### LDL

As shown in the **Figure 7H**, the network meta-analysis demonstrated that all drugs that act as hypoglycemic agents had insignificant effects on LDL levels in patients with NAFLD with or without type 2 diabetes. Compared with placebo, only gliclazide and sitagliptin achieved a better effect than the placebo, and all other drugs were less effective than the placebo. SUCRA showed that gliclazide, sitagliptin and placebo had the strongest treatment effects with probabilities of 55%, 42% and 40%. Liraglutide and ipragliflozin had the weakest effects, with 74% and 63% probabilities, respectively, of being ranked among the bottom three (**Figure 6H**). The Ranking probability of different hypoglycemic agents was shown in **Figure 5H**. The specific results of the comparison of the effects of different drugs on LDL levels are shown in the **Supplementary Materials** (**Table S9**).

### Weight

As shown in the **Figure 5I**, in terms of body weight and BMI, pioglitazone carries a significantly higher risk of weight gain in NAFLD patients with or without type 2 diabetes than do other drugs, and the probability that its therapeutic effect ranks among the bottom three is as high as 99%. Liraglutide and exenatide can significantly reduce the weight of patients with NAFLD with or without type 2 diabetes, and the probabilities that their therapeutic effects rank among the top three are 98% and 78%, respectively (**Figure 6I**). The results of the network meta-analysis showed that except for pioglitazone and gliclazide, hypoglycemic agents were better than placebo in improving the weight of NAFLD patients with or without type 2 diabetes (**Figure 7I**). The specific results of the comparison of the effects of different drugs on body weight are shown in the **Supplementary Materials** (**Table 10**).

### Network Heterogeneity and Inconsistency

The difference in DIC between the consistency model and the inconsistency model was less than 5, indicating that the data basically met the premise of consistency. The global heterogeneity of the mesh meta-analysis results was determined by I^2^ and p value. The **Supplementary Materials** showed that the I^2^ of all outcome indicators was less than 5%. In addition, all the different outcome indicators were found to have P values > 0.05 by the global consistency test, indicating low global heterogeneity (**Supplementary Materials** **Table S11**, **12**). In the base case analysis of baseline changes in all outcome indicators, the RE model provided better fitting in terms of DIC and mean posterior residuals than the FE model except for body weight and HDL levels (**Supplementary Materials** **Table S11**). The network inconsistency in different outcome indicators was evaluated by means of a node-splitting approach based on Monte Carlo Markov chain simulations considering random-effect models, normal priors for treatment fixed effects, and uniform priors for the variances of the random effects. The analysis (**Supplementary Materials** **Table S13**) indicated that local inconsistency did not exist except for the following: (1) ALT for sitagliptin versus placebo (p=0.043); (2) BMI for metformin versus liraglutide, pioglitazone versus liraglutide and pioglitazone versus metformin (p=0.028, 0.044, and 0.017, respectively); (3) HDL for metformin versus liraglutide (p=0.011); and (4) LDL for pioglitazone versus metformin (p=0.033).

Through convergence analysis, it can be found that each MCM chain has reached stable fusion from the initial part, and it can be visually analyzed in the subsequent calculation. The fluctuation of a single chain cannot be recognized, so the degree of convergence is higher. The trace map, density map and convergence diagnosis diagram of each outcome index are shown in the **Supplementary Materials** (**Figures S1–S9**).

## Discussion

Nonalcoholic fatty liver disease is a type of metabolic stress liver injury. Its pathogenesis is very complex, and the diagnosis and treatment of NAFLD has always been clinically difficult. The spectrum of disease includes nonalcoholic simple fatty liver (NAFL), nonalcoholic steatohepatitis (NASH), and associated cirrhosis and HCC (37, 38). Currently, NAFLD patients account for approximately one-quarter of the world’s total population, while recent data released by Chinese scholars show that the incidence of NAFLD in China is as high as 29.2% (39). At present, internationally renowned expert groups in gastroenterology, liver disease, nutrition and pathology have reached a consensus that it is appropriate to change the designation NAFLD to “metabolic-related fatty liver disease (MAFLD)” (40).

We compared the therapeutic effects of various hypoglycemic drugs on specific metabolic indexes of NAFLD patients through a network meta-analysis. Through analysis, we found that although thiazolidinedione hypoglycemic agents, especially pioglitazone, carry the highest risk of increasing patients’ weight and BMI compared to other drugs, they have a good therapeutic effect on other metabolic indicators. In a recent network meta-analysis, pioglitazone, a representative thiazolidinedione, were also found to be most effective in reducing the nonalcoholic fatty liver disease activity score, this is consistent with our results and indicates the reliability of our results (41). In 2015, a network meta-analysis of patients with NASH showed that thiazolidinediones are superior to placebo in improving key histological features in NASH (42), but that study only compared thiazolidinediones with other drugs such as vitamin E and pentoxifylline, and the efficacies of different hypoglycemic drugs were not compared. However, some scholars have found that pioglitazone can improve disproportionate body fat distribution, which is important for the maintenance of cardiometabolic health; therefore, we believe that treatment with pioglitazone combined with other glucose-lowering drugs to improve body weight and BMI may be one of the better choices for the treatment of NAFLD (43).

As the first GLP-1 RA drug approved for the treatment of type 2 diabetes, exenatide was shown in our study to have obvious advantages in improving body weight and BMI, but it is not as effective as liraglutide in improving triglyceride and HDL levels, which are also affected by GLP-1 RAs. However, due to the lack of randomized controlled trials on exenatide, well-designed, placebo-controlled, double-blind studies with histology-proven outcomes are needed to evaluate the effect of exenatide on NAFLD.

Another GLP-1 RA, liraglutide, has a poor therapeutic effect on LDL, and the possibility of its ranking among the bottom three is also high. However, liraglutide has a good therapeutic effect on other metabolic indicators. It can significantly improve body weight and BMI. In a meta-analysis that included six randomized controlled trials, treatment with liraglutide for 26 weeks at 1.8 mg/day improved ALT levels and liver steatosis in 4442 T2DM patients (44). Therefore, liraglutide appears to be the most promising GLP-1RA for the prevention and/or treatment of NAFLD/NASH, although a large number of randomized controlled trials are needed to confirm its effect (45).

Metformin has always been recommended as a first-line drug for the treatment of T2DM. The results of our analysis show that in addition to improving the weight and BMI of patients, the therapeutic effects of metformin on other indicators are moderate. Meta-analysis shows that metformin can improve liver function, insulin resistance and body mass index (BMI) to a certain extent, but it does not affect the histological response of NAFLD patients (46). Therefore, current guidelines do not recommend the use of metformin for adult NASH (47, 48). According to our analysis and the abovementioned research, metformin may be beneficial to NAFLD patients, especially those with T2DM, but its specific effect still has a certain gap compared with GPP1 RAs.

As a first-class DPP-4 inhibitor, our results show that sitagliptin is only effective at improving triglyceride and ALT levels and that it has no significant effect on other indicators. However, the conclusions of different scholars regarding the effect of sitagliptin on nonalcoholic fatty liver disease are also contradictory. Studies have shown that sitagliptin significantly reduces the lipid content of the liver and total body fat. In another study, sitagliptin did not reduce liver enzyme levels after 12 months of treatment, although HbA1c levels were reduced by 0.7%. These observations are also consistent with our results (49, 50).

SGLT2 inhibitors represent a new type of hypoglycemic agent that can improve hyperglycemia by inhibiting the reabsorption of sugar by the kidneys. The results of our analysis show that, in addition to lowering blood sugar, ipragliflozin also has a good effect on weight loss, BMI and triglycerides. As another SGLT2 inhibitor, dapagliflozin has better benefits in weight loss but displays no significant advantages in other areas. Therefore, based on our analysis, we believe that SGLT2 inhibitors should not currently be recommended for NAFLD treatment. Of course, because few related studies were included in the analysis, a large-scale placebo-controlled randomized controlled trial is still needed to verify our conclusions.

### Strengths and Limitations of This Study

NAFLD is a recent research hotspot, and there are still no specific guidelines for a plan of treatment for patients with NAFLD. There are currently no approved drugs for the treatment of NAFLD, although many hypoglycemic agents have been tested in NAFLD patients. To date, no scholar has compared and analyzed the therapeutic effects of all hypoglycemic drugs on NAFLD patients. Therefore, for the first time, we used a network meta-analysis to comprehensively analyze the therapeutic effects of all currently used hypoglycemic drugs on glucose metabolism and liver biological indicators in NAFLD patients. However, our research has certain limitations. First, a significant proportion of patients with NAFLD have normal liver function, making these tests insufficient as a valid biomarker for the entire NAFLD spectrum. Choosing these markers as primary endpoints through which to evaluate the effectiveness of hypoglycemic agents in treating NAFLD was an important simplification. Second, the randomized controlled trials we included in this study tended to comprise very different patient populations with different therapeutic backgrounds. Some of the included randomized controlled trials had small sample sizes. In addition, most of the studies included in our meta-analysis were about pioglitazone, liraglutide and metformin. These facts affect the reliability of our results to some extent. For other drugs, because there are fewer randomized controlled trials, there are fewer studies included in the analysis, resulting in larger confidence intervals for some research results and weaker strength of the evidence presented. Unlike a recent network analysis, we also did not include lifestyle interventions and surgical operations, mainly because we considered the lack of RCTs in the field of bariatric surgery and the small number of patients enrolled in lifestyle modification trials. Therefore, a large-scale randomized controlled trial of related drugs is still needed to verify our conclusions. Second, due to the limited number of studies in the meta-analysis, we conducted a comprehensive analysis of NAFLD patients with or without diabetes without subgroup analysis, and this may affect the accuracy of our results to a certain extent.

## Conclusion

In conclusion, bearing in mind the limitations mentioned above, thiazolidinediones, GLP1 RAs and metformin (in particular, pioglitazone) may be the most promising therapeutic approaches for the treatment of NAFLD, but they carry a risk of significant weight gain. We believe that GLP1 RA drugs (such as liraglutide and exenatide) or metformin can be used in combination in clinical practice to offset the risk of weight gain caused by thiazolidinedione drugs. Regarding the efficacy of other drugs in NAFLD patients and the efficacy of thiazolidinedione drugs in combination with GLP1 RA drugs or metformin, long-term, large-scale placebo-controlled, rigorous randomized controlled studies are still required.

## Data Availability Statement

The original contributions presented in the study are included in the article/**Supplementary Material**. Further inquiries can be directed to the corresponding author.

## Author Contributions

JL participated in the conception and design of this research, evaluated the data, and critically revised the manuscript. JF and JL read and approved the final version. JF and JL participated in data collection, collation and manuscript writing. JF and JL are the guarantors of this work and are responsible for the completeness of the data and the accuracy of the data analysis. All authors contributed to the article and approved the submitted version.

## Funding

This research was funded by the National Natural Science Foundation of China(81670736).

## Conflict of Interest

The authors declare that the research was conducted in the absence of any commercial or financial relationships that could be construed as a potential conflict of interest.
