# Supplementary material for: Efficacy of Various Hypoglycemic Agents in the Treatment of Patients With Nonalcoholic Liver Disease With or Without Diabetes： A Network Meta-Analysis
Source: Front Endocrinol (Lausanne). 2021 Mar 24;12:649018. doi: 10.3389/fendo.2021.649018 (PMC8024567; doi:10.3389/fendo.2021.649018)

**Appendix 1: Search strategy for identifying RCTs assessing the effects of different anti-diabetic drugs in the treatment of non-alcoholic liver disease patients with or without diabetes**

**Cochrane：**

ID Search

#1 (non-alcoholic fatty liver disease):ti,ab,kw OR (NAFLD):ti,ab,kw

#2 metformin:ti,ab,kw

#3 Thiazolidinedione:ti,ab,kw

#4 Exenatide:ti,ab,kw

#5 Liraglutide:ti,ab,kw

#6 Lixisenatide:ti,ab,kw

#7 Dulaglutide:ti,ab,kw

#8 Semaglutide:ti,ab,kw

#9 Tirzepatide:ti,ab,kw

#10 Sitagliptin:ti,ab,kw

#11 Vildagliptin:ti,ab,kw

#12 Dapagliflozin:ti,ab,kw

#13 Canagliflozin:ti,ab,kw

#14 Empagliflozin:ti,ab,kw

#15 Ipragliflozin:ti,ab,kw

#16 #3 or #4 or #5 or #6 or #7 or #8 or #9 or #10 or #11 pr #12 or #13 or #14 or #15

#17 #2 or #3 or #4 or #5 or #6 or #7 or #8 or #9 or #10 or #11 pr #12 or #13 or #14 or #15

#18 #1 and #17

**PubMed**

1. NAFLD

2. non-alcoholic fatty liver disease

3. (non-alcoholic fatty liver disease) OR (NAFLD)

4. Metformin

5. Thiazolidinediones

6. Exenatide

7. Liraglutide

8. Lixisenatide

9. Dulaglutide

10. Semaglutide

11. Tirzepatide

12. Sitagliptin

13. Dapagliflozin

14. Canagliflozin

15. Empagliflozin

16. Ipragliflozin

17. Vildagliptin

18. (((((((((((((Vildagliptin) OR (Ipragliflozin)) OR (Empagliflozin)) OR (Canagliflozin)) OR (Dapagliflozin)) OR (Sitagliptin)) OR (Tirzepatide)) OR (Semaglutide)) OR (Dulaglutide)) OR (Lixisenatide)) OR (Liraglutide)) OR (Exenatide)) OR (Thiazolidinediones)) OR (METFORMIN)

19. ((((((((((((((Vildagliptin) OR (Ipragliflozin)) OR (Empagliflozin)) OR (Canagliflozin)) OR (Dapagliflozin)) OR (Sitagliptin)) OR (Tirzepatide)) OR (Semaglutide)) OR (Dulaglutide)) OR (Lixisenatide)) OR (Liraglutide)) OR (Exenatide)) OR (Thiazolidinediones)) OR (METFORMIN)) AND ((non-alcoholic fatty liver disease) OR (NAFLD))

| Table s1 Main characteristics of RCTs Included in the Network Meta-Analysis | | | | | | | | | | | | | | | | | |
| --- | --- | --- | --- | --- | --- | --- | --- | --- | --- | --- | --- | --- | --- | --- | --- | --- | --- |
| study | **Drug** | **setting** | **total**  **N** | **Male**  **%** | **Mean**  **Age in**  **years** | **Study Type**  **and**  **Follow-up weeks** | **BMI** | **Weight (kg)** | **FPG (mg/dL)** | **HbA1c (%)** | **HDL** | **LDL** | **Triglyceride level** | | **Plasma AST** | **Plasma ALT** | |
| Cusi 2016 | Pioglitazone | single center，USA | 50 | 36(72.0%) | 52(10) | Parallel, 18month | 34.3(4.8) | 98.2(16.5) | 125(27.1) | 6.4(1.0) | 36(9) | 109(44) | 224(171) | 47(21) | | | 62(33) |
|  | Placebo |  | 51 | 35(68.6%) | 49(11) |  | 34.5(4.8) | 99.2(17.0) | 122(27.1) | 6.3(1.0) | 37(9) | 109(33) | 179(109) | 43(22) | | | 57(33) |
| Belfort 2006 | Pioglitazone  Placebo | single center，USA | 26  21 | 14(53.8%)  7(33.3%) | 51(7)  51±10 | Parallel, 28 weeks | 33.5(4.9)  32.9(4.4) | 93.7(18.1)  90.2(15.4) | 119(35)  115(28) | 6.2(1.5)  6.2(1.1) | 40(9)  37(9) | 118(31)  117(37) | 156(87)  173(142) | 47(15)  42(16) | | | 67(26)  61(33) |
| Bril 2019 | Pioglitazone | multicenter，USA | 37 | 30(81.0%) | 60(6) | Parallel, 18 months | 35.2(4.3) | NR | 144(43) | 7.3(1.1) | 38(10) | 91(44) | 163(42) | 32(18) | | | 40(25) |
|  | Placebo |  | 36 | 33(91.7%) | 60(9) |  | 33.8 (4.6) | NR | 158(41) | 7.5(1.3) | 39(9) | 98(39) | 156(21) | 41(22) | | | 53(32) |
| Yan 2015 | Pioglitazone | multicenter, China | 60 | 32(51.6% | 53.5(8.6) | Parallel, 16 weeks | 27.5(3.7) | 75(12.7) | NR | 6.4(0.7) | 46(10) | 122(35) | 191(81) | 28(17) | | | 41(29) |
|  | Placebo |  | 62 | 28(46.7%) | 50.6(10.7) |  | 27.3(2.8) | 75.7(11.1) | NR | 6.2(0.7) | 46(10) | 109(26) | 171(62) | 25(7) | | | 34(25) |
| Feng 2019 | Liraglutide | single center, China | 29 | 21(72.4%) | 47(10) | Parallel, 24 months | 28.1(3.2) | 81(12) | 158.4(43.2) | 8.9(1.7) | 38(11) | 97(29) | 241(118) | 31(14) | | | 50(31) |
|  | Metformin |  | 29 | 19(65.5%) | 46(12) |  | 26.8(3.8) | 75(13) | 144(34.2) | 9.4(1.8) | 45(23) | 109(29) | 217(118) | 34(17) | | | 51(32) |
|  | Gliclazide |  | 27 | 19(70.4%) | 48(13) |  | 27.5 (2.6) | 78(13) | 162(28.8) | 9.1(1.2) | 43(10) | 113(26) | 253(151) | 27(11) | | | 42(26) |
| Yan 2019 | Liraglutide | multicenter trial, China | 24 | 17(70.8%) | 43.1(9.7) | Parallel, 28 weeks | 30.1(3.3) | 86.6(12.9) | 154.8(50.4) | 7.8(1.4) | 43(8) | 104(31) | 204(97) | 31.1(11.7) | | | 43.2(21.2) |
|  | Sitagliptin |  | 27 | 21(77.8%) | 45.7(9.2) |  | 29.7(2.8) | 88.2(13.6) | 151.2(45) | 7.6(0.9) | 46(23) | 120(27) | 230(124) | 34.4(16.9) | | | 46.0(25.5) |
| Shimizu 2018 | Dapagliflozin | single center, Japan | 33 | 19(57.6%) | 56.2(11.5) | Parallel, 24 weeks | 27.6(4.7) | 73.9(16.1) | 137.9(54.1) | 6.35(1.5) | 50(13) | 105(34) | 133(55) | 28(22) | | | 38(29) |
|  | Placebo |  | 24 | 15(62.5%) | 57.1(13.8) |  | 28.3(3.5) | 76.4(13.9) | 136.62(41.22) | 6.6(0.6) | 46(12) | 102(24) | 145(89) | 30(13) | | | 33(16) |
| Ito 2017 | Pioglitazone | multicenter, Japan | 34 | 18(52.9%) | 59.1(9.8) | Parallel, 24 weeks | 29.9(6.2) | 76.7(15.2) | 169.4(50.9) | 8.3(1.4) | 47.4(11.6) | 104.0(27.9) | 188.4(148.8) | 43.3(20.5) | | | 53.1(26.6) |
|  | Ipragliflozin |  | 32 | 14(43.8%) | 57.3(12.1) |  | 30.7(5.0) | 79.6(17.9) | 160.1(38.7) | 8.5(1.5) | 48.9(9.3) | 108.3(36.2) | 166.9(76.4) | 39.7(16.7） | | | 57.4(27.3) |
| Eriksson 2018 | Placebo | multicenter, Sweden | 21 | 17(81%) | 65.6 (6.1) | Parallel, 12 weeks | 30.3 (3.1) | 93.0 (12.2) | 169.2(29.7) | 7.4(0.8) | 51(15) | 98(34) | 169(85) | 29.4(13.2) | | | 34.2(12.6) |
|  | Dapagliflozin |  | 21 | 16(76.2%) | 65.0 (6.5) |  | 30.5 (2.8) | 90.2 (8.7) | 161.8(33.3) | 7.4(0.6) | 50(10) | 110(35) | 178(104) | 31.2 (11.4) | | | 40.2(15) |
| Smits 2016 | Liraglutide | single center, Netherlands | 17 | 12(70.6%) | 61(7) | Parallel, 12 weeks | 32.8(4.1) | 103(13) | 149.4(21.6) | 7.4(0.8) | NR | NR | NR | 24.2 (8) | | | 29(12) |
|  | Sitagliptin |  | 17 | 14(82.4%) | 62(7) |  | 31.4(4.5) | 99(18) | 142.2(14.4) | 7.1(0.4) | NR | NR | NR | 22.8(6) | | | 29(13) |
|  | Placebo |  | 17 | 13(76.5%) | 66(6) |  | 30.6(2.9) | 96(10) | 160.2(36) | 7.5(0.8) | NR | NR | NR | 22.2 (7) | | | 32(21) |
| Cui 2017 | Sitagliptin | single center, USA | 25 | 13(52%) | 52.9(11.9) | Parallel, 24 weeks | 31.9(5.4) | 92.8(18.7) | 104.0(27.0) | 6.1(0.5) | NR | 100.0 (42.0) | 185.0 (52.0) | 28.0 (15.0) | | | 43.0 (26.0) |
|  | Placebo |  | 25 | 8(32%) | 54.9(11.3) |  | 31.7(4.7) | 86.3(20.6) | 106.0(38.5) | 6.2(0.8) | NR | 89.5 (46.0) | 150.0 (96.0) | 29.0 (19.0) | | | 40.0 (26.0) |
| Sofer 2016 | metformin | single center, Israel | 32 | 17(53.1%) | 51.9(10.9) | Parallel, 4 months | 32.6(5.8) | NR | 135.0(52.8) | NR | 41.3(12.2) | 103.5(37.3) | 195.4(119.1) | 29.0(18.0) | | | 38.5(31.7) |
|  | Placebo |  | 31 | 14(45.2%) | 55.2(14.0) |  | 31.5(5.6) | NR | 98.1(15.9) | NR | 47.8(14.3) | 110.8(35.2) | 140.8(58.0) | 29.4(8.4) | | | 34.9(15.8) |
| Sanyal 2010 | Placebo | multicenter, USA | 83 | 25(30.1%) | 45.4(11.2) | Parallel, 96 weeks | 35(7) | 99(21) | 95(14) | NR | 43(11) | 125(35) | 165(89) | 55(30) | | | 81(48) |
|  | Pioglitazone |  | 80 | 21(26.3%) | 47.0(12.6) |  | 34(6) | 97(23) | 92(12) | NR | 45(12) | 120(31) | 162(84) | 54(26) | | | 82(45) |
| fan 2013 | Exenatide | single center, China | 49 | 28(57.1%) | 51.02 (10.10) | Parallel, 12 weeks | 28.2(1.8) | 80.6(11.2) | 152.1(11.5) | 8.1(0.5) | 41(9) | 99(32) | 219(132) | 35.9(12.3) | | | 65.7(18.1) |
|  | Metformin |  | 68 | 38(55.9%) | 54.68(12.14) |  | 27.6(1.7) | 78.6(10.8) | 151.4(12.4) | 8.1(0.6) | 38(8) | 101(32) | 222(153) | 34.3(13.7) | | | 65.8(17.6) |
| haukeland 2009 | Placebo | single center, Norway | 24 | 16(66.7%) | 49.9 (12.8) | Parallel, 6 months | 30.3 (3.3) | 91.5 (12.1) | 102.6(18) | 5.8 (0.4) | 48(10) | 128(35) | 177(133) | NR | | | NR |
|  | Metformin |  | 20 | 6(30%) | 44.3 (9.0) |  | 31.4 (3.9) | 97.1 (17.6) | 99(14.4) | 5.7 (0.6) | 48(14) | 151(35) | 159(71) | NR | | | NR |
| Nar A 2009 | Placebo | single center, USA | 31 | 20(64.5%) | 47.6(9.0) | Parallel, 48 weeks | 33.6(4.1) | 98.0(18.9) | 100.5(16.0) | NR | NR | NR | NR | 54.7(24.2) | | | 89.5(51.6) |
|  | metformin |  | 37 | 19(51.4%) | 49.7 (10.4) |  | 33.1(6.4) | 94.7(20.9) | 106.7(33.6) | NR | NR | NR | NR | 52.0(37.1) | | | 73.8(64.1) |
| Armstrong 2016 | Liraglutide | multicenter, UK | 26 | 11(42.3%) | 50 (11) | Parallel, 48 weeks | 34.2(4.7) | 101 (18) | 108(30.6) | 5.9 (0.7) | 43(15) | 101(31) | 168(97) | 51 (22) | | | 77 (34) |
|  | Placebo |  | 26 | 13(50%) | 52 (12) |  | 37.7(6.2) | 108 (18) | 109.8(27) | 6.0(0.9) | 50(8) | 112(39) | 159(71) | 51 (27) | | | 66 (42) |
| Anushiravani 2019 | placebo | single center, Iran | 30 | 66(73.3%) | 47.0(9.1) | Parallel, 3 months | 26.1(3.1) | NR | NR | NR | 58.4(8.6) | 131.2(48.8) | 150.2(54.2) | 19.6(11.7) | | | 22.8(15.9) |
|  | pioglitazone |  | 30 |  |  |  | 25.1(3.7) | NR | NR | NR | 48.5(9.1) | 113.7(34.3) | 155.1(83.7) | 23.3(11.1) | | | 30.2(18.1) |
|  | metformin |  | 30 |  |  |  | 25(3.3) | NR | NR | NR | 63.3(15.1) | 96.6(35.4) | 125.6(67.2) | 28.4(13.6) | | | 42.3(22.6) |
| Omer 2010 | Metformin | single center, Turkey | 22 | 7(31.8%) | 48.0(9.8) | Parallel, 12 months | 30.8(6.6) | NR | 118.7(23) | 5.8(1.3) | 41.1(10) | 117.4(47) | 149.7(55) | 46.1(24) | | | 63.09(24) |
|  | Rosiglitazone |  | 20 | 11(55%) | 49.3(6) |  | 28.4(4.1) | NR | 126.3(26) | 6.0(0.7) | 43.5(12) | 140.5(22) | 188.9(95) | 53.8(41) | | | 64.9(23) |
| Yaghoubi 2017 | placebo | single center, Iran | 30 | NR | 38(10) | Parallel, 12 weeks | 27.0(4.0) | NR | 94(15) | NR | NR | NR | 151(32) | 45(12) | | | 67(21) |
|  | pioglitazone |  | 30 | NR | 35(7) |  | 26.0(3.0) | NR | 107(20) | NR | NR | NR | 164(35) | 61(30) | | | 76(30) |
| Hajiaghamohammadi 2012 | Pioglitazone | single center, Iran | 22 | NR | 32.6(6.4) | Parallel,2 months | 27.4(1.7) | 76.9(9.2) | 95.45(6.88) | NR | NR | NR | 252.2(52.8) | 55.0(9.5) | | | 77.45(18.5) |
|  | Metformin |  | 22 | NR |  |  | 27.5(1.9) | 77.0(9.5) | 95.09(7.00) | NR | NR | NR | 248.4(53.2) | 54.9(11.3) | | | 78.36(19.9) |
| Joy2017 | Sitagliptin | single center, UK | 6 | 3 (50%) | 56.7(9.9) | Parallel, 24 weeks | 35.9(6.6) | 100.4(28.7) | NR | 7.9(1.0) | 43.3(17.8) | 55(37.5) | 247.8(145.1) | 44(22) | | | 72(50) |
|  | Placebo |  | 6 | 2 (33%) | 54.7(9.8) |  | 37.4(4.7) | 105.8(23.5) | NR | 8.2(0.9) | 42.6(13.2) | 62.3(27.4) | 206.2(177) | 39(19) | | | 46(36) |
| Razavizade2013 | Metformin | single center, Iran | 40 | 31(77.5%) | 36.4(9.0) | Parallel, 4 months | 27.9(2.3) | 80.3(5.9) | 94.5(18.9) | NR | 51.41(14.7) | 95.5(31.6) | 152.8(4.8) | 49.1(16.8) | | | 87.7(44.9) |
|  | Pioglitazone |  | 40 | 37(92.5%) | 34.2(6.8) |  | 27.5(3.9) | 83.5(12.3) | 97.4(12.9) | NR | 49.33(10.3) | 101.3(18.7) | 137.8(90.6) | 50.5(21.8) | | | 66.0(56.8) |
| Tian2018 | liraglutide | single center, China | 52 | 31(59.6%) | 58.5(7.6) | Parallel, 12 weeks | 28.1(1.9) | 76.9(10.0) | 152.1(11.5) | 8.1(0.5) | 40.6(9.3) | 95.2(20.5) | 218.6(131.9) | 36.0(12.3) | | | 66.74(18.1) |
|  | Metformin |  | 75 | 43(57.3%) | 56.4(8.4) |  | 27.6(1.8) | 76.3(10) | 151.4(12.4) | 8.1(0.6) | 38.3(7.7) | 101.4(31.7) | 222.1(153.1) | 34.3(13.7) | | | 65.8(17.6) |
| Zhang2020 | Liraglutide | single center, China | 30 | 13(43.3%) | 50.2(11.5) | Parallel, 24 weeks | 27.6(5.2) | 79.3(8.8) | 160.2(52.2) | 8.1(2.0) | 42.6(7.7) | 127.7(38.7) | 79.7(53.1) | 33.1(11.6) | | | 30(10.4) |
|  | Pioglitazone |  | 30 | 15(50%) | 51 .5(12.1) |  | 27.1(3.8) | 78.0(9.2) | 156.6(59.4) | 8.1(1.7) | 42.6(15.5) | 127.7(50.3) | 79.7(53.1) | 33.1(16.3) | | | 36.6(24.7) |
| Han2020 | Placebo | single center, China | 15 | 9(60%) | 56.7(11.8) | Parallel, 24 weeks | 30.2(2.5) | 81.4(8.5) | 118.4(19.7) | 6.6(0.6) | 50.0(11.9) | 91.3(16.9) | 152.5(91.8) | 30.4(19.6) | | | 31.1(13.5) |
|  | Ipragliflozin |  | 30 | 19(63.3%) | 52.5(10.3) |  | 30.6(5.3) | 84.3(17.2) | 121.2(21.3) | 6.7(0.7) | 50.7(13.2) | 102.8(28.9) | 159.7(66.9) | 26.6(13.0) | | | 33.4(25.1) |

**The data are presented as mean (standard deviation); BMI：body mass index; FPG: fasting plasma glucose; HbA1c: glycosylated hemoglobin; HDL: high density lipoprotein; LDL: low density lipoprotein; AST: alanine aminotransferase ; ALT: alanine aminotransferase; NR: not report**

| Table s2 Network analysis results of comparison between different drugs in ALT | | | | | | | | | | |
| --- | --- | --- | --- | --- | --- | --- | --- | --- | --- | --- |
| Dapagliflozin | -5.54 (-27.32, 16.09) | 15.84 (-6.08, 37.92) | 3.12 (-14.27, 19.56) | -0.46 (-15.06, 14.65) | 6.07 (-8.51, 20.51) | 0.87 (-12.72, 14.22) | 8.88 (-3, 20.89) | -13.4 (-38.58, 11.69) | 2.66 (-13.53, 19.05) |  |
| 5.54 (-16.09, 27.32) | **Exenatide** | 21.36 (-2.4, 45.37) | 8.68 (-13.02, 29.61) | 5.03 (-13.11, 23.81) | 11.6 (-4.43, 27.72) | 6.43 (-11.75, 24.48) | 14.43 (-3.48, 32.57) | -7.85 (-33.93, 18.18) | 8.17 (-12.04, 28.83) |  |
| -15.84 (-37.92, 6.08) | -21.36 (-45.37, 2.4) | **Gliclazide** | -12.76  (-34.84, 8.46) | -16.3 (-33.84, 1.44) | -9.78 (-27.56, 7.8) | -14.97 (-33.75, 3.37) | -6.94 (-25.5, 11.45) | -29.26 (-56.47, -2.39) | -13.19 (-33.31, 7.03) |  |
| -3.12 (-19.56, 14.27) | -8.68 (-29.61, 13.02) | 12.76 (-8.46, 34.84) | **Ipragliflozin** | -3.62 (-17.33, 11.63) | 2.92 (-10.6, 17.3) | -2.26 (-14.17, 10.35) | 5.73 (-5.7, 18.21) | -16.48 (-41.03, 8.62) | -0.49 (-16.04, 16.24) |  |
| 0.46 (-14.65, 15.06) | -5.03 (-23.81, 13.11) | 16.3 (-1.44, 33.84) | 3.62 (-11.63, 17.33) | **Liraglutide** | 6.51 (-2.92, 15.47) | 1.34 (-8.26, 10.25) | 9.36 (0.34, 18) | -12.96 (-35.79, 9.27) | 3.11 (-7.95, 13.99) |  |
| -6.07 (-20.51, 8.51) | -11.6 (-27.72, 4.43) | 9.78 (-7.8, 27.56) | -2.92 (-17.3, 10.6) | -6.51 (-15.47, 2.92) | **Metformin** | -5.17 (-13.55, 2.97) | 2.84 (-5.28, 11.09) | -19.47 (-40.08, 0.99) | -3.39 (-15.87, 9.3) |  |
| -0.87 (-14.22, 12.72) | -6.43 (-24.48, 11.75) | 14.97 (-3.37, 33.75) | 2.26 (-10.35, 14.17) | -1.34 (-10.25, 8.26) | 5.17 (-2.97, 13.55) | **Pioglitazone** | 8.01 (2.02, 14.3) | -14.29 (-36.32, 7.79) | 1.8 (-10.05, 14.02) |  |
| -8.88 (-20.89, 3) | -14.43 (-32.57, 3.48) | 6.94 (-11.45, 25.5) | -5.73 (-18.21, 5.7) | -9.36 (-18, -0.34) | -2.84 (-11.09, 5.28) | -8.01 (-14.3, -2.02) | **Placebo** | -22.31 (-44.48, -0.34) | -6.22 (-17.29, 4.9) |  |
| 13.4 (-11.69, 38.58) | 7.85 (-18.18, 33.93) | 29.26 (2.39, 56.47) | 16.48 (-8.62, 41.03) | 12.96 (-9.27, 35.79) | 19.47 (-0.99, 40.08) | 14.29 (-7.79, 36.32) | 22.31 (0.34, 44.48) | **Rosiglitazone** | 16.11 (-7.92, 40.33) |  |
| -2.66 (-19.05, 13.53) | -8.17 (-28.83, 12.04) | 13.19 (-7.03, 33.31) | 0.49 (-16.24, 16.04) | -3.11 (-13.99, 7.95) | 3.39 (-9.3, 15.87) | -1.8 (-14.02, 10.05) | 6.22 (-4.9, 17.29) | -16.11 (-40.33, 7.92) | **Sitagliptin** |  |

**The reported results are displayed with effect size and 95% confidence interval (95% CI). Weighted mean difference (WMD) is applied to continuous results.**

| Table s3 Network analysis results of comparison between different drugs in AST | | | | | | | | | | |
| --- | --- | --- | --- | --- | --- | --- | --- | --- | --- | --- |
| Dapagliflozin | -6.97 (-21.23, 6.93) | 3.97 (-9.36, 17.22) | 3.05 (-8.71, 13.65) | -2.15 (-11.88, 7.53) | 0.61 (-8.98, 9.9) | -2.09  (-11.21, 6.71) | 2.99  (-5, 10.99) | 0.46  (-26.36, 26.83) | -2.01  (-12.77, 8.24) |  |
| 6.97 (-6.93, 21.23) | **Exenatide** | 10.93 (-3.43, 25.59) | 10.01 (-4.17, 23.45) | 4.83 (-6.9, 16.88) | 7.58 (-2.88, 18.05) | 4.87  (-6.7, 16.51) | 9.95  (-1.46, 21.71) | 7.45 (-19.49, 34.08) | 4.97  (-8.14, 17.84) |  |
| -3.97 (-17.22, 9.36) | -10.93 (-25.59, 3.43) | **Gliclazide** | -0.94 (-14.46, 11.62) | -6.11 (-16.12, 3.88) | -3.34 (-13.59, 6.63) | -6.05  (-16.88, 4.53) | -0.97  (-11.6, 9.69) | -3.49  (-30.42, 23.01) | -5.96  (-17.91, 5.57) |  |
| -3.05 (-13.65, 8.71) | -10.01 (-23.45, 4.17) | 0.94 (-11.62, 14.46) | **Ipragliflozin** | -5.18 (-14.02, 4.64) | -2.43  (-11.01, 6.88) | -5.13  (-12.79, 3.27) | -0.06  (-7.27, 8.36) | -2.47  (-28.87, 23.77) | -5.04  (-15.01, 5.46) |  |
| 2.15 (-7.53, 11.88) | -4.83 (-16.88, 6.9) | 6.11 (-3.88, 16.12) | 5.18 (-4.64, 14.02) | **Liraglutide** | 2.75  (-3.04, 8.31) | 0.04  (-5.9, 5.79) | 5.14  (-0.37, 10.69) | 2.6  (-23.04, 27.83) | 0.13  (-6.93, 6.79) |  |
| -0.61 (-9.9, 8.98) | -7.58 (-18.05, 2.88) | 3.34 (-6.63, 13.59) | 2.43 (-6.88, 11.01) | -2.75 (-8.31, 3.04) | **Metformin** | -2.69  (-7.72, 2.34) | 2.39  (-2.49, 7.55) | -0.1  (-25.08, 24.48) | -2.62  (-10.47, 5.07) |  |
| 2.09 (-6.71, 11.21) | -4.87 (-16.51, 6.7) | 6.05 (-4.53, 16.88) | 5.13 (-3.27, 12.79) | -0.04 (-5.79, 5.9) | 2.69  (-2.34, 7.72) | **Pioglitazone** | 5.08  (1.22, 9.21) | 2.58  (-22.88, 27.69) | 0.08  (-7.51, 7.46) |  |
| -2.99 (-10.99, 5) | -9.95 (-21.71, 1.46) | 0.97 (-9.69, 11.6) | 0.06 (-8.36, 7.27) | -5.14  (-10.69, 0.37) | -2.39  (-7.55, 2.49) | -5.08  (-9.21, -1.22) | **Placebo** | -2.53  (-28.05, 22.5) | -5.02  (-12.06, 1.63) |  |
| -0.46 (-26.83, 26.36) | -7.45  (-34.08, 19.49) | 3.49 (-23.01, 30.42) | 2.47 (-23.77, 28.87) | -2.6 (-27.83, 23.04) | 0.1  (-24.48, 25.08) | -2.58  (-27.69, 22.88) | 2.53  (-22.5, 28.05) | R**osiglitazone** | -2.54  (-28.24, 23.6) |  |
| 2.01 (-8.24, 12.77) | -4.97 (-17.84, 8.14) | 5.96 (-5.57, 17.91) | 5.04 (-5.46, 15.01) | -0.13 (-6.79, 6.93) | 2.62  (-5.07, 10.47) | -0.08  (-7.46, 7.51) | 5.02  (-1.63, 12.06) | 2.54  (-23.6, 28.24) | **Sitagliptin** |  |

**The reported results are displayed with effect size and 95% confidence interval (95% CI). Weighted mean difference (WMD) is applied to continuous results.**

| Table s4 Network analysis results of comparison between different drugs in Triglycerides | | | | | | | | |  |
| --- | --- | --- | --- | --- | --- | --- | --- | --- | --- |
| Dapagliflozin | -10.91  (-67.72, 47.26) | -57.21  (-125.01, 9.57) | -40.13  (-67.85, -9.93) | -31.98  (-61.69, -2.06) | -18.67  (-46.25, 11.02) | -28.75  (-53.6, -3.64) | -18.68  (-41.35, 5.18) | -59.6  (-117.92, -3.61) | |
| 10.91  (-47.26, 67.72) | **Exenatide** | -46.66  (-127.08, 32.6) | -28.87  (-84.53, 26.17) | -20.99  (-76.69, 33.49) | -7.88  (-57.38, 41.88) | -17.95  (-71.22, 34.13) | -7.61  (-60.73, 44.58) | -49.18  (-125.06, 24.56) | |
| 57.21  (-9.57, 125.01) | 46.66  (-32.6, 127.08) | **Gliclazide** | 17.29  (-46.97, 83.7) | 25.43  (-35.96, 87.6) | 39.1  (-22.98, 101.55) | 28.48  (-33.93, 92.17) | 38.69  (-24.03, 102.75) | -2.31  (-87.56, 81.11) | |
| 40.13  (9.93, 67.85) | 28.87  (-26.17, 84.53) | -17.29  (-83.7, 46.97) | **Ipragliflozin** | 7.88  (-19.8, 35.04) | 21.27  (-2.73, 45.94) | 11.21  (-9.92, 30.11) | 21.25  (2.95, 38.89) | -19.93  (-76.61, 34.34) | |
| 31.98  (2.06, 61.69) | 20.99  (-33.49, 76.69) | -25.43  (-87.6, 35.96) | -7.88  (-35.04, 19.8) | **Liraglutide** | 13.62  (-9.8, 36.76) | 3.27  (-17.96, 23.69) | 13.4  (-8.01, 34.84) | -28.19  (-85, 27.88) | |
| 18.67  (-11.02, 46.25) | 7.88  (-41.88, 57.38) | -39.1  (-101.55, 22.98) | -21.27  (-45.94, 2.73) | -13.62  (-36.76, 9.8) | **Metformin** | -10.18  (-27.53, 6.39) | -0.1  (-17.87, 17.13) | -41.38  (-97.22, 12.48) | |
| 28.75  (3.64, 53.6) | 17.95  (-34.13, 71.22) | -28.48  (-92.17, 33.93) | -11.21  (-30.11, 9.92) | -3.27  (-23.69, 17.96) | 10.18  (-6.39, 27.53) | **Pioglitazone** | 10.11  (0.07, 21.05) | -31.22  (-85.06, 21.54) | |
| 18.68  (-5.18, 41.35) | 7.61  (-44.58, 60.73) | -38.69  (-102.75, 24.03) | -21.25  (-38.89, -2.95) | -13.4  (-34.84, 8.01) | 0.1  (-17.13, 17.87) | -10.11  (-21.05, -0.07) | **Placebo** | -41.28  (-94.4, 10.1) | |
| 59.6  (3.61, 117.92) | 49.18  (-24.56, 125.06) | 2.31  (-81.11, 87.56) | 19.93  (-34.34, 76.61) | 28.19  (-27.88, 85) | 41.38  (-12.48, 97.22) | 31.22  (-21.54, 85.06) | 41.28  (-10.1, 94.4) | **Sitagliptin** | |

**The reported results are displayed with effect size and 95% confidence interval (95% CI). Weighted mean difference (WMD) is applied to continuous results.**

| Table s5 Network analysis results of comparison between different drugs in BMI | | | | | | | | | |
| --- | --- | --- | --- | --- | --- | --- | --- | --- | --- |
| Dapagliflozin | -3.18  (-6.33, 0) | -0.4  (-3.68, 2.86) | -1.78  (-4.77, 1.21) | -2.07  (-4.94, 0.74) | -1.58  (-4.36, 1.25) | -0.4  (-3.15, 2.36) | -0.98  (-3.64, 1.69) | 1.31  (-2.94, 5.63) | -1.19  (-4.28, 1.88) |
| 3.18  (0, 6.33) | **Exenatide** | 2.79  (0.37, 5.09) | 1.41  (-0.85, 3.6) | 1.14  (-0.76, 2.8) | 1.61  (0.11, 3.11) | 2.8  (1.03, 4.49) | 2.21  (0.44, 3.92) | 4.49  (0.97, 8.06) | 2  (-0.27, 4.17) |
| 0.4  (-2.86, 3.68) | -2.79  (-5.09, -0.37) | **Gliclazide** | -1.39  (-3.71, 0.99) | -1.66  (-3.49, 0.1) | -1.17  (-2.97, 0.69) | 0.01  (-1.92, 1.96) | -0.58  (-2.49, 1.36) | 1.71  (-1.93, 5.46) | -0.79  (-3.08, 1.52) |
| 1.78  (-1.21, 4.77) | -1.41  (-3.6, 0.85) | 1.39  (-0.99, 3.71) | **Ipragliflozin** | -0.26  (-2.04, 1.31) | 0.2  (-1.4, 1.88) | 1.39  (-0.16, 2.93) | 0.8  (-0.58, 2.19) | 3.1  (-0.48, 6.72) | 0.6  (-1.48, 2.62) |
| 2.07  (-0.74, 4.94) | -1.14  (-2.8, 0.76) | 1.66  (-0.1, 3.49) | 0.26  (-1.31, 2.04) | **Liraglutide** | 0.48  (-0.4, 1.54) | 1.66  (0.69, 2.76) | 1.07  (0.17, 2.1) | 3.38  (0.06, 6.79) | 0.87  (-0.6, 2.41) |
| 1.58  (-1.25, 4.36) | -1.61  (-3.11, -0.11) | 1.17  (-0.69, 2.97) | -0.2  (-1.88, 1.4) | -0.48  (-1.54, 0.4) | **Metformin** | 1.18  (0.29, 2.02) | 0.59  (-0.31, 1.46) | 2.89  (-0.32, 6.12) | 0.38  (-1.28, 2) |
| 0.4  (-2.36, 3.15) | -2.8  (-4.49, -1.03) | -0.01  (-1.96, 1.92) | -1.39  (-2.93, 0.16) | -1.66  (-2.76, -0.69) | -1.18  (-2.02, -0.29) | **Pioglitazone** | -0.59  (-1.29, 0.13) | 1.71  (-1.61, 5.07) | -0.79  (-2.44, 0.83) |
| 0.98  (-1.69, 3.64) | -2.21  (-3.92, -0.44) | 0.58  (-1.36, 2.49) | -0.8  (-2.19, 0.58) | -1.07  (-2.1, -0.17) | -0.59  (-1.46, 0.31) | 0.59  (-0.13, 1.29) | **Placebo** | 2.3  (-1.02, 5.66) | -0.2  (-1.76, 1.32) |
| -1.31  (-5.63, 2.94) | -4.49  (-8.06, -0.97) | -1.71  (-5.46, 1.93) | -3.1  (-6.72, 0.48) | -3.38  (-6.79, -0.06) | -2.89  (-6.12, 0.32) | -1.71  (-5.07, 1.61) | -2.3  (-5.66, 1.02) | **Rosiglitazone** | -2.51  (-6.15, 1.07) |
| 1.19  (-1.88, 4.28) | -2  (-4.17, 0.27) | 0.79  (-1.52, 3.08) | -0.6  (-2.62, 1.48) | -0.87  (-2.41, 0.6) | -0.38  (-2, 1.28) | 0.79  (-0.83, 2.44) | 0.2  (-1.32, 1.76) | 2.51  (-1.07, 6.15) | **Sitagliptin** |

**The reported results are displayed with effect size and 95% confidence interval (95% CI). Weighted mean difference (WMD) is applied to continuous results.**

| Table s6 Network analysis results of comparison between different drugs in FPG | | | | | | | | | |
| --- | --- | --- | --- | --- | --- | --- | --- | --- | --- |
| Dapagliflozin | -4.35  (-34.82, 22.01) | -7.45  (-38.39, 19.87) | 5.35  (-20.76, 28.06) | -13.61  (-38.29, 7.01) | 0.04  (-22.41, 19.33) | -4.21  (-26.05, 14.61) | 8.04  (-11.58, 25.41) | -24.39  (-57.21, 5.09) | -6.91  (-32.5, 15.69) |
| 4.35  (-22.01, 34.82) | **Exenatide** | -2.98  (-31.89, 25.49) | 9.74  (-16.71, 36.43) | -8.93  (-32.68, 12.75) | 4.5  (-15.04, 24.03) | 0.25  (-21.62, 22.29) | 12.45  (-8.74, 34.71) | -19.92  (-50.21, 10.38) | -2.4  (-28.06, 23.15) |
| 7.45  (-19.87, 38.39) | 2.98  (-25.49, 31.89) | **Gliclazide** | 12.73  (-14.06, 40.06) | -6.06  (-28.33, 15.05) | 7.48  (-13.33, 28.62) | 3.24  (-19.11, 26.05) | 15.54  (-6.35, 38.37) | -16.92  (-48.14, 14.44) | 0.6  (-24.81, 26.14) |
| -5.35  (-28.06, 20.76) | -9.74  (-36.43, 16.71) | -12.73  (-40.06, 14.06) | **Ipragliflozin** | -18.76  (-39.31, -0.24) | -5.22  (-23.31, 12.51) | -9.48  (-25.98, 6.95) | 2.73  (-12.74, 19.01) | -29.7  (-59.01, -0.64) | -12.16  (-34.02, 9.44) |
| 13.61  (-7.01, 38.29) | 8.93  (-12.75, 32.68) | 6.06  (-15.05, 28.33) | 18.76  (0.24, 39.31) | **Liraglutide** | 13.51  (2.63, 26.02) | 9.28  (-2.79, 23.23) | 21.64  (10.34, 34.87) | -10.87  (-36.03, 15.81) | 6.68  (-8.34, 22.83) |
| -0.04  (-19.33, 22.41) | -4.5  (-24.03, 15.04) | -7.48  (-28.62, 13.33) | 5.22  (-12.51, 23.31) | -13.51  (-26.02, -2.63) | **Metformin** | -4.26  (-14, 5.78) | 8.01  (-0.98, 17.94) | -24.41  (-47.51, -1.27) | -6.89  (-23.42, 9.62) |
| 4.21  (-14.61, 26.05) | -0.25  (-22.29, 21.62) | -3.24  (-26.05, 19.11) | 9.48  (-6.95, 25.98) | -9.28  (-23.23, 2.79) | 4.26  (-5.78, 14) | **Pioglitazone** | 12.25  (4.41, 20.82) | -20.18  (-45.42, 4.86) | -2.63  (-19.4, 13.82) |
| -8.04  (-25.41, 11.58) | -12.45  (-34.71, 8.74) | -15.54  (-38.37, 6.35) | -2.73  (-19.01, 12.74) | -21.64  (-34.87, -10.34) | -8.01  (-17.94, -0.98) | -12.25  (-20.82, -4.41) | **Placebo** | -32.45  (-57.85, -7.87) | -14.94  (-30.48, -0.08) |
| 24.39 (-5.09, 57.21) | 19.92  (-10.38, 50.21) | 16.92  (-14.44, 48.14) | 29.7  (0.64, 59.01) | 10.87  (-15.81, 36.03) | 24.41  (1.27, 47.51) | 20.18  (-4.86, 45.42) | 32.45  (7.87, 57.85) | **Rosiglitazone** | 17.55  (-10.98, 45.88) |
| 6.91  (-15.69, 32.5) | 2.4  (-23.15, 28.06) | -0.6  (-26.14, 24.81) | 12.16  (-9.44, 34.02) | -6.68  (-22.83, 8.34) | 6.89  (-9.62, 23.42) | 2.63  (-13.82, 19.4) | 14.94  (0.08, 30.48) | -17.55  (-45.88, 10.98) | **Sitagliptin** |

**The reported results are displayed with effect size and 95% confidence interval (95% CI). Weighted mean difference (WMD) is applied to continuous results.**

| Table s7 Network analysis results of comparison between different drugs in HbA1c | | | | | | | | | |
| --- | --- | --- | --- | --- | --- | --- | --- | --- | --- |
| Dapagliflozin | -0.15  (-1.42, 1.02) | 0.36  (-0.89, 1.53) | 0.14  (-0.78, 1.05) | -0.25  (-1.1, 0.53) | -0.21  (-1.15, 0.63) | 0.04  (-0.77, 0.77) | 0.34  (-0.32, 0.97) | -0.41  (-1.77, 0.86) | -0.03  (-0.94, 0.78) |
| 0.15  (-1.02, 1.42) | **Exenatide** | 0.51  (-0.74, 1.76) | 0.29  (-0.88, 1.55) | -0.09  (-1.09, 0.91) | -0.06  (-0.89, 0.77) | 0.2  (-0.89, 1.3) | 0.49  (-0.51, 1.56) | -0.26  (-1.53, 1.02) | 0.12  (-1.01, 1.24) |
| -0.36  (-1.53, 0.89) | -0.51  (-1.76, 0.74) | **Gliclazide** | -0.22  (-1.38, 1.01) | -0.61  (-1.54, 0.33) | -0.58  (-1.51, 0.36) | -0.32  (-1.38, 0.76) | -0.02  (-1.02, 1.02) | -0.77  (-2.12, 0.57) | -0.39  (-1.5, 0.7) |
| -0.14  (-1.05, 0.78) | -0.29  (-1.55, 0.88) | 0.22  (-1.01, 1.38) | **Ipragliflozin** | -0.39  (-1.23, 0.37) | -0.36  (-1.28, 0.48) | -0.1  (-0.81, 0.54) | 0.2  (-0.46, 0.84) | -0.56  (-1.9, 0.72) | -0.17  (-1.07, 0.63) |
| 0.25  (-0.53, 1.1) | 0.09  (-0.91, 1.09) | 0.61  (-0.33, 1.54) | 0.39  (-0.37, 1.23) | **Liraglutide** | 0.03  (-0.53, 0.58) | 0.29  (-0.3, 0.88) | 0.59  (0.12, 1.11) | -0.17  (-1.29, 0.94) | 0.22  (-0.42, 0.84) |
| 0.21  (-0.63, 1.15) | 0.06  (-0.77, 0.89) | 0.58  (-0.36, 1.51) | 0.36  (-0.48, 1.28) | -0.03  (-0.58, 0.53) | **Metformin** | 0.26  (-0.44, 0.97) | 0.56  (-0.03, 1.2) | -0.2  (-1.16, 0.76) | 0.18  (-0.58, 0.94) |
| -0.04  (-0.77, 0.77) | -0.2  (-1.3, 0.89) | 0.32  (-0.76, 1.38) | 0.1  (-0.54, 0.81) | -0.29  (-0.88, 0.3) | -0.26  (-0.97, 0.44) | **Pioglitazone** | 0.3  (-0.09, 0.73) | -0.45  (-1.66, 0.73) | -0.07  (-0.76, 0.59) |
| -0.34  (-0.97, 0.32) | -0.49  (-1.56, 0.51) | 0.02  (-1.02, 1.02) | -0.2  (-0.84, 0.46) | -0.59  (-1.11, -0.12) | -0.56  (-1.2, -0.03) | -0.3  (-0.73,-0.09) | **Placebo** | -0.76  (-1.93, 0.36) | -0.37  (-0.96, 0.15) |
| 0.41  (-0.86, 1.77) | 0.26  (-1.02, 1.53) | 0.77  (-0.57, 2.12) | 0.56 (  -0.72, 1.9) | 0.17  (-0.94, 1.29) | 0.2  (-0.76, 1.16) | 0.45  (-0.73, 1.66) | 0.76  (-0.36, 1.93) | **Rosiglitazone** | 0.38  (-0.85, 1.61) |
| 0.03  (-0.78, 0.94) | -0.12  (-1.24, 1.01) | 0.39  (-0.7, 1.5) | 0.17  (-0.63, 1.07) | -0.22  (-0.84, 0.42) | -0.18  (-0.94, 0.58) | 0.07  (-0.59, 0.76) | 0.37  (-0.15, 0.96) | -0.38  (-1.61, 0.85) | **Sitagliptin** |

**The reported results are displayed with effect size and 95% confidence interval (95% CI). Weighted mean difference (WMD) is applied to continuous results.**

| Table s8 Network analysis results of comparison between different drugs in HDL | | | | | | | | |
| --- | --- | --- | --- | --- | --- | --- | --- | --- |
| Dapagliflozin | 0.3  (-4.58, 5.18) | 1.85  (-4.52, 8.24) | 1.93  (-0.97, 4.8) | 2.98  (-0.76, 6.67) | 2.29  (-1.45, 6.01) | 2.39  (-0.71, 5.47) | -0.49  (-3.27, 2.28) | -0.08  (-5.66, 5.51) |
| -0.3  (-5.18, 4.58) | **Exenatide** | 1.55  (-4.87, 7.96) | 1.63  (-2.45, 5.68) | 2.68  (-1.26, 6.6) | 1.99  (-1.13, 5.12) | 2.08  (-1.98, 6.15) | -0.79  (-4.79, 3.19) | -0.37  (-6.15, 5.37) |
| -1.85  (-8.24, 4.52) | -1.55  (-7.96, 4.87) | **Gliclazide** | 0.07  (-5.75, 5.86) | 1.12  (-4.17, 6.41) | 0.44  (-5.2, 6.05) | 0.53  (-5.32, 6.33) | -2.34  (-8.12, 3.38) | -1.93  (-8.73, 4.83) |
| -1.93  (-4.8, 0.97) | -1.63  (-5.68, 2.45) | -0.07  (-5.86, 5.75) | **Ipragliflozin** | 1.05  (-1.53, 3.63) | 0.37  (-2.23, 2.97) | 0.46  (-1.04, 1.98) | -2.42  (-3.18, -1.66) | -2.01  (-6.88, 2.9) |
| -2.98  (-6.67, 0.76) | -2.68  (-6.6, 1.26) | -1.12  (-6.41, 4.17) | -1.05  (-3.63, 1.53) | **Liraglutide** | -0.68  (-3.08, 1.69) | -0.6  (-3.23, 2.04) | -3.47  (-5.93, -1) | -3.06  (-7.31, 1.22) |
| -2.29  (-6.01, 1.45) | -1.99  (-5.12, 1.13) | -0.44  (-6.05, 5.2) | -0.37  (-2.97, 2.23) | 0.68  (-1.69, 3.08) | **Metformin** | 0.09  (-2.49, 2.69) | -2.78  (-5.27, -0.3) | -2.37  (-7.22, 2.49) |
| -2.39  (-5.47, 0.71) | -2.08  (-6.15, 1.98) | -0.53  (-6.33, 5.32) | -0.46  (-1.98, 1.04) | 0.6  (-2.04, 3.23) | -0.09  (-2.69, 2.49) | **Pioglitazone** | -2.88  (-4.21, -1.54) | -2.47  (-7.39, 2.47) |
| 0.49  (-2.28, 3.27) | 0.79  (-3.19, 4.79) | 2.34  (-3.38, 8.12) | 2.42  (1.66, 3.18) | 3.47  (1.00, 5.93) | 2.78  (0.3, 5.27) | 2.88  (1.54, 4.21) | **Placebo** | 0.41  (-4.41, 5.26) |
| 0.08  (-5.51, 5.66) | 0.37  (-5.37, 6.15) | 1.93  (-4.83, 8.73) | 2.01  (-2.9, 6.88) | 3.06  (-1.22, 7.31) | 2.37  (-2.49, 7.22) | 2.47  (-2.47, 7.39) | -0.41  (-5.26, 4.41) | **Sitagliptin** |

**The reported results are displayed with effect size and 95% confidence interval (95% CI). Weighted mean difference (WMD) is applied to continuous results.**

| Table s9 Network analysis results of comparison between different drugs in LDL | | | | | | | | |
| --- | --- | --- | --- | --- | --- | --- | --- | --- |
| Dapagliflozin | -0.37  (-24.58, 22.92) | -3.68  (-28.02, 20.53) | 6.35  (-13.2, 22.68) | 3.77  (-14.13, 20.96) | -1.43  (-18.24, 14.95) | -1.55  (-16.02, 14.04) | -1.09  (-14.19, 12.76) | -1.04  (-22.43, 19.78) |
| 0.37  (-22.92, 24.58) | **Exenatide** | -3.19  (-28.48, 22.09) | 6.62  (-17.06, 28.11) | 4.16  (-15.81, 23.98) | -1.04  (-18.2, 16.06) | -1.14  (-20.01, 19.58) | -0.72  (-19.5, 19.8) | -0.68  (-24.82, 23.48) |
| 3.68  (-20.53, 28.02) | 3.19  (-22.09, 28.48) | **Gliclazide** | 9.76  (-14.16, 31.71) | 7.3  (-11.33, 25.68) | 2.14  (-16.52, 20.68) | 2.1  (-17.77, 23.03) | 2.58  (-17.15, 23.21) | 2.48  (-21.21, 26.38) |
| -6.35  (-22.68, 13.2) | -6.62  (-28.11, 17.06) | -9.76  (-31.71, 14.16) | **Ipragliflozin** | -2.5  (-17.32, 14.46) | -7.73  (-21.28, 8.2) | -8  (-18.38, 7.26) | -7.71  (-17.19, 7.09) | -7.19  (-26.26, 13.5) |
| -3.77  (-20.96, 14.13) | -4.16  (-23.98, 15.81) | -7.3  (-25.68, 11.33) | 2.5  (-14.46, 17.32) | **Liraglutide** | -5.2  (-15.2, 5.06) | -5.24  (-16.36, 7.41) | -4.81  (-15.44, 7.24) | -4.78  (-20.97, 11.33) |
| 1.43  (-14.95, 18.24) | 1.04  (-16.06, 18.2) | -2.14  (-20.68, 16.52) | 7.73  (-8.2, 21.28) | 5.2  (-5.06, 15.2) | **Metformin** | -0.08  (-9.39, 10.7) | 0.37  (-8.75, 10.68) | 0.41  (-16.75, 17.48) |
| 1.55  (-14.04, 16.02) | 1.14  (-19.58, 20.01) | -2.1  (-23.03, 17.77) | 8  (-7.26, 18.38) | 5.24  (-7.41, 16.36) | 0.08  (-10.7, 9.39) | **Pioglitazone** | 0.46  (-6.5, 7.12) | 0.48  (-17.44, 17.1) |
| 1.09  (-12.76, 14.19) | 0.72  (-19.8, 19.5) | -2.58  (-23.21, 17.15) | 7.71  (-7.09, 17.19) | 4.81  (-7.24, 15.44) | -0.37  (-10.68, 8.75) | -0.46  (-7.12, 6.5) | **Placebo** | 0.04  (-16.91, 15.88) |
| 1.04  (-19.78, 22.43) | 0.68  (-23.48, 24.82) | -2.48  (-26.38, 21.21) | 7.19  (-13.5, 26.26) | 4.78  (-11.33, 20.97) | -0.41  (-17.48, 16.75) | -0.48  (-17.1, 17.44) | -0.04  (-15.88, 16.91) | **Sitagliptin** |

**The reported results are displayed with effect size and 95% confidence interval (95% CI). Weighted mean difference (WMD) is applied to continuous results.**

| Table s10 Network analysis results of comparison between different drugs in Weight | | | | | | | | |
| --- | --- | --- | --- | --- | --- | --- | --- | --- |
| Dapagliflozin | -1.87  (-6.61, 2.9) | 3.86  (-2.7, 10.41) | 0.19  (-1.03, 1.4) | -2.16  (-4.42, 0.1) | 1.31  (-1.49, 4.09) | 5.8  (3.99, 7.6) | 2.18  (1.01, 3.36) | 0.38  (-4.74, 5.51) |
| 1.87  (-2.9, 6.61) | **Exenatide** | 5.73  (-1.72, 13.16) | 2.05  (-2.58, 6.65) | -0.3  (-4.87, 4.25) | 3.17  (-0.71, 7.02) | 7.66  (3.07, 12.25) | 4.04  (-0.57, 8.64) | 2.24  (-4.38, 8.84) |
| -3.86  (-10.41, 2.7) | -5.73  (-13.16, 1.72) | **Gliclazide** | -3.68  (-10.15, 2.8) | -6.02  (-12.3, 0.24) | -2.57  (-8.9, 3.78) | 1.93  (-4.54, 8.44) | -1.68  (-8.14, 4.79) | -3.49  (-11.4, 4.45) |
| -0.19  (-1.4, 1.03) | -2.05  (-6.65, 2.58) | 3.68  (-2.8, 10.15) | **Ipragliflozin** | -2.34  (-4.3, -0.39) | 1.12  (-1.42, 3.67) | 5.61  (4.21, 7.03) | 2  (1.66, 2.33) | 0.2  (-4.8, 5.21) |
| 2.16  (-0.1, 4.42) | 0.3  (-4.25, 4.87) | 6.02  (-0.24, 12.3) | 2.34  (0.39, 4.3) | **Liraglutide** | 3.46  (1.03, 5.89) | 7.95  (5.84, 10.07) | 4.34  (2.41, 6.27) | 2.54  (-2.35, 7.45) |
| -1.31  (-4.09, 1.49) | -3.17  (-7.02, 0.71) | 2.57  (-3.78, 8.9) | -1.12  (-3.67, 1.42) | -3.46  (-5.89, -1.03) | **Metformin** | 4.49  (2, 6.99) | 0.87  (-1.65, 3.4) | -0.93  (-6.27, 4.43) |
| -5.8  (-7.6, -3.99) | -7.66  (-12.25, -3.07) | -1.93  (-8.44, 4.54) | -5.61  (-7.03, -4.21) | -7.95  (-10.07, -5.84) | -4.49  (-6.99, -2) | **Pioglitazone** | -3.62  (-4.99, -2.25) | -5.42  (-10.52, -0.31) |
| -2.18  (-3.36, -1.01) | -4.04  (-8.64, -0.57) | 1.68  (-4.79, 8.14) | -2  (-2.33, -1.66) | -4.34  (-6.27, -2.41) | -0.87  (-3.4, 1.65) | 3.62  (2.25, 4.99) | **Placebo** | -1.8  (-6.79, 3.19) |
| -0.38  (-5.51, 4.74) | -2.24  (-8.84, 4.38) | 3.49  (-4.45, 11.4) | -0.2  (-5.21, 4.8) | -2.54  (-7.45, 2.35) | 0.93  (-4.43, 6.27) | 5.42  (0.31, 10.52) | 1.8  (-3.19, 6.79) | **Sitagliptin** |

**The reported results are displayed with effect size and 95% confidence interval (95% CI). Weighted mean difference (WMD) is applied to continuous results.**

| Table s11: Model fit statistics for all outcomes | | | | | | | |
| --- | --- | --- | --- | --- | --- | --- | --- |
| Outcome | **Model** | **DIC** | **Dbar** | **pD** | **ratio** | **I^2** | **Used in base case analyses** |
| ALT | FE | 110.22 | 76.25 | 33.96 | 1.439 | 32% | No |
|  | RE | 98.33 | 53.43 | 44.90 | 1.008 | 3% | Yes |
| AST | FE | 86.81 | 54.80 | 32.00 | 1.118 | 12% | No |
|  | RE | 97.91 | 52.69 | 45.22 | 0.994 | 0% | Yes |
| Triglyceride | FE | 81.59 | 50.61 | 30.98 | 1.054 | 7% | No |
|  | RE | 83.46 | 49.71 | 33.75 | 1.036 | 5% | Yes |
| Weight | FE | 70.85 | 40.86 | 29.99 | 0.888 | 0% | Yes |
|  | RE | 72.50 | 39.73 | 32.77 | 0.864 | 0% | No |
| BMI | FE | 86.81 | 54.80 | 32.01 | 1.118 | 12% | No |
|  | RE | 83.26 | 45.27 | 37.99 | 0.924 | 0% | Yes |
| FPG | FE | 109.86 | 77.85 | 32.01 | 1.622 | 40% | No |
|  | RE | 91.80 | 49.70 | 42.10 | 1.035 | 5% | Yes |
| HbA1c (%) | FE | 95.80 | 67.77 | 28.02 | 1.694 | 42% | No |
|  | RE | 74.12 | 38.59 | 35.53 | 0.965 | 0% | Yes |
| LDL cholesterol | FE | 81.14 | 52.11 | 29.03 | 1.184 | 17% | No |
|  | RE | 79.26 | 44.04 | 35.23 | 1.001 | 2% | Yes |
| HDL cholesterol | FE | 68.29 | 40.27 | 27.99 | 0.959 | 0% | Yes |
|  | RE | 69.76 | 38.97 | 30.79 | 0.928 | 0% | No |
| DIC, deviance information criterion; FE, fixed effects; RE, random effects; BMI：body mass index; FPG: fasting plasma glucose; HbA1c: glycosylated hemoglobin; HDL: high density lipoprotein; LDL: low density lipoprotein; AST: alanine aminotransferase ; ALT: alanine aminotransferase. | | | | | | | |
|  | | | | | | | |

| Table s12 Heterogeneity assessment in network | | | |
| --- | --- | --- | --- |
| outcomes | **numbers of trails** | **number of participants** | **heterogeneity(p)** |
| ALT | 25 | 1768 | 0.6905 |
| AST | 25 | 1768 | 0.4353 |
| Triglycerides | 23 | 1651 | 0.3268 |
| BMI | 23 | 1641 | 0.1955 |
| FPG | 23 | 1588 | 0.923 |
| HbA1c | 20 | 1244 | 0.8952 |
| HDL | 20 | 1497 | 0.2908 |
| LDL | 21 | 1547 | 0.0675 |
| Weight | 22 | 1557 | 0.6274 |

**P value for all comparisons was used to evaluate the global inconsistency of different indicators, if P>0.05, it means that the overall consistency of the data is better.**

| Table s13 Assessment of local inconsistencies in different outcome indicators | | | | | | |
| --- | --- | --- | --- | --- | --- | --- |
| **Outcome indicators** | **Comparison** | **Direct WMD (95%CI)** | **Indirect WMD (95%CI)** | **Network**  **WMD (95%CI)** | **P-value** |  |
| **ALT** | Ipragliflozin vs Pioglitazone | 2.9  (-16.0, 22.0) | -5.5  (-23.0, 11.0) | -2.3  (-14.0, 10.0) | 0.48 |  |
|  | Ipragliflozin vs Placebo | 3.1  ( -13.0, 19.0) | 12.0  (-8.6, 32.) | 5.7  (-5.7, 18.0) | 0.48 |  |
|  | Liraglutide vs Metformin | 8.1  (-5.4, 21.0) | 4.3  (-9.3, 18.0) | 6.5  (-2.9, 15.0) | 0.67 |  |
|  | Liraglutide vs Pioglitazone | 9.9  (-6.9, 27.0) | -2.3  (-13.0, 8.3) | 1.3  (-8.2, 10.0) | 0.23 |  |
|  | Liraglutide vs Placebo | 3.7  (-10.0,19.0) | 13.0  (0.65, 23.0) | 9.4  (0.4, 18.0) | 0.34 |  |
|  | Liraglutide vs Sitagliptin | -1.8  (-13.0, 9.0) | 15.0  (-5.6, 34.0) | 3.1  (-7.9, 14.0) | 0.145 |  |
|  | Metformin vs Pioglitazone | -5.5  ( -18.0, 6.1) | -5.3  (-19.0, 8.4) | -5.2  (-13.0, 3.0) | 0.98 |  |
|  | Metformin vs Placebo | 6.8  (-5.2, 18.0) | -1.6  (-14.0, 11.0) | 2.9  (-5.2, 11.0) | 0.31 |  |
|  | Pioglitazone vs Placebo | 9.8  (3.1, 17.0) | 2.0  (-9.3, 15.0) | 8.1  (2.0, 14.0) | 0.26 |  |
|  | Placebo vs Sitagliptin | 1.7  (-10.0, 13.0) | -22.0  (-41.0, -2.7) | -6.3  (-17.0, 4.9) | **0.04** |  |
| **AST**  **AST** | Ipragliflozin vs Pioglitazone | 1.5  ( -11.0, 14.0) | -9.1  (-19.0, 0.9) | -5.1  ( -13.0, 3.3) | 0.17 |  |
|  | Ipragliflozin vs Placebo | -3.4  ( -13.0, 6.0) | 7.3  (-5.3, 20.0) | -0.04  (-7.3, 8.3) | 0.16 |  |
|  | Liraglutide vs Metformin | 2.8  (-5.6, 11.0) | 2.6  (-6.0, 11.0) | 2.7  (-3.1, 8.3) | 0.97 |  |
|  | Liraglutide vs Pioglitazone | 5. 0  (-6.6, 17.0) | -1.7  (-8.7, 5.0) | 0.04  (-5.9, 5.8) | 0.3 |  |
|  | Liraglutide vs Placebo | 3.6  (-5.0, 13.0) | 6.2  (-1.0, 13.0) | 5.1  (-0.3, 11.0) | 0.63 |  |
|  | Liraglutide vs Sitagliptin | -1.1  (-9.5, 6.8) | 1.4  ( -13.0, 15.0) | 0.1  (-7.0, 6.8) | 0.75 |  |
|  | Metformin vs Pioglitazone | -2.7  (-9.9, 4.4) | -2.8  (-11.0, 5.6) | -2.7  (-7.7, 2.4) | 0.98 |  |
|  | Metformin vs Placebo | 3.3  (-4.2, 11.0) | 1.5  (-6.0, 9.5) | 2.4  (-2.5, 7.6) | 0.72 |  |
|  | Pioglitazone vs Placebo | 6.7  (2.2, 11.) | 0.5  (-7.0, 8.5) | 5.1  (1.2, 9.2) | 0.18 |  |
|  | Placebo vs Sitagliptin | -1.8  (-9.9, 5.4) | -15.0  ( -28.0, -1.1) | -5.0  (-12.0, 1.6) | 0.11 |  |
| **Triglyceride** | Dapagliflozin vs Liraglutide | 16.0  (-49.0, 81.0) | -44.0  (-78.0, -11.0) | 32. 0  (-61.0, -1.5) | 0.11 |  |
|  | Dapagliflozin vs Placebo | -25.0  (-49.0, -0.8) | 36.0  (-34.0, 150.1) | -18. 0  (-41.0, 4.7) | 0.11 |  |
|  | Ipragliflozin vs Pioglitazone | 5.5  (-53.0, 63.0) | 12.0  (-12.0, 34.0) | 11.0  (-9.4, 30.0) | 0.84 |  |
|  | Ipragliflozin vs Placebo | 22.0  (1.1, 43.0) | 14.0  (-43.0, 72.0) | 21.0  (3.4, 38.0) | 0.80 |  |
|  | Liraglutide vs Metformin | 16.0  (-22.0, 56.0) | 12.0  (-19.0, 41.0) | 14.0  (-10.0, 37.0) | 0.85 |  |
|  | Liraglutide vs Pioglitazone | 9.0  (-20.0, 38.0) | -3.6  (-35.0, 27.0) | 3.7  (-18.0, 23.0) | 0.54 |  |
|  | Liraglutide vs Placebo | 19.0  (-33.0, 70.0) | 13.0  (-110.0, 36.0) | 14.0  (-8.2, 34.0) | 0.81 |  |
|  | Metformin vs Pioglitazone | -2.1  (-24.0, 20.0) | -27.0  (-61.0, 4.7) | -10.0  (-27.0, 6.2) | 0.20 |  |
|  | Metformin vs Placebo | -10.0  (-37.0, 15.0) | 8.6  (-16.0, 34.0) | 0.0  (-17.0, 17.0) | 0.29 |  |
|  | Pioglitazone vs Placebo | 13.0  (1.8, 26.0) | -9.5  (-41.0, 21.0) | 10.0  (0.0, 21.0) | 0.18 |  |
| BMI | Liraglutide vs Metformin | 0.22  (-1.1, 0.83) | 1.6  (0.26, 2.9) | 0.47  (-0.38, 1.5) | **0.03** |  |
|  | Liraglutide vs Pioglitazone | 4.1  (1.7, 6.6) | 1.3  (0.24, 2.3) | 1.7  (0.70, 2.8) | **0.04** |  |
|  | Liraglutide vs Placebo | 1.4  (0.03, 2.8) | 0.65  (-0.55, 2.2) | 1.1  (0.18, 2.1) | 0.42 |  |
|  | Liraglutide vs Sitagliptin | 0.61  (-1.2, 2.4) | 1.8  (-1.2, 5.0) | 0.87  (-0.63, 2.4) | 0.5 |  |
|  | Metformin vs Pioglitazone | 0.55  (-0.38, 1.50) | 2.5  (1.2, 3.7) | 1.20  (0.29, 2.0) | **0.02** |  |
|  | Metformin vs Placebo | 0.46  (-0.95, 1.9) | 0.73  (-0.50, 1.9) | 0.60  (-0.31, 1.5) | 0.77 |  |
|  | Pioglitazone vs Placebo | -0.64  (-1.4, 0.19) | -0.27  (-2.1, 1.3) | -0.59  (-1.3, 0.12) | 0.70 |  |
|  | Placebo vs Sitagliptin | -0.043  (-2.1, 2.1) | -0.46  (-3.1, 1.9) | -0.21  (-1.8, 1.3) | 0.79 |  |
| **FPG**  **FPG** | Ipragliflozin vs Pioglitazone | -6.9  (-34.0, 21.0) | -11.0  (-34.0, 11.0) | -9.5  (-26.0, 6.9) | 0.80 |  |
|  | Ipragliflozin vs Placebo | 1.6  (-19.0, 22.0) | 5.8  (-23.0, 35.0) | 2.7  (-13.0, 19.0) | 0.80 |  |
|  | Liraglutide vs Metformin | 8.8  (-5.0, 24.0) | 21.0  (3.3, 39.0) | 13.0  (2.7, 26.0) | 0.28 |  |
|  | Liraglutide vs Pioglitazone | 23.0  (-10.0, 57.0) | 7.1  (-6.2, 22.0) | 9.2  (-2.8, 23.0) | 0.38 |  |
|  | Liraglutide vs Placebo | 29.0  (9.8, 48.0) | 18.0  (4.2, 35.0) | 22.0  (10.0, 35.0) | 0.36 |  |
|  | Liraglutide vs Sitagliptin | 4.4  ( -15.0, 24.0) | 17.0  (-12.0, 47.0) | 6.6  (-8.1, 23.0) | 0.47 |  |
|  | Metformin vs Pioglitazone | -5.2  (-20.0, 9.7) | -3.1  (-18.0, 12.0) | -4.3  (-14.0, 5.7) | 0.83 |  |
|  | Metformin vs Placebo | 5.3  (-7.8, 20.0) | 11.0  (-2.8, 25.0) | 8.0  (-1.1, 18.0) | 0.56 |  |
|  | Pioglitazone vs Placebo | 13.0  (3.2, 24.0) | 10.0  (-5.3, 26.0) | 12.0  (4.4, 21.0) | 0.74 |  |
|  | Placebo vs Sitagliptin | -15.0  (-34.0, 4.2) | -14.0  (-51.0,21.0) | -15.0  (-30.0, -0.1) | 0.96 |  |
| **HbA1c (%)** | Ipragliflozin vs Pioglitazone | -0.30  (-1.3, 0.73) | 0.03  (-0.97, 0.97) | -0.10  (-0.81, 0.54) | 0.61 |  |
|  | Ipragliflozin vs Placebo | 0.30  (-0.56, 1.2) | -0.03  (-1.1, 1.1) | 0.20  (-0.47, 0.84) | 0.61 |  |
|  | Liraglutide vs Metformin | -0.12  (-0.83, 0.54) | 0.41  (-0.62, 1.5) | 0.033  (-0.53, 0.58) | 0.35 |  |
|  | Liraglutide vs Pioglitazone | 0.39  (-0.82, 1.6) | 0.25  (-0.46, 0.97) | 0.29  (-0.3, 0.89) | 0.84 |  |
|  | Liraglutide vs Placebo | 0.80  (0.12, 1.5) | 0.39  (-0.38, 1.2) | 0.59  (0.12, 1.1) | 0.41 |  |
|  | Liraglutide vs Sitagliptin | 0.29  (-0.55, 1.1) | 0.26  (-0.75, 1.2) | 0.22  (-0.42, 0.84) | 0.96 |  |
|  | Metformin vs Placebo | 0.30  (-0.60, 1.2) | 0.83  (-0.016, 1.8) | 0.55  (-0.024, 1.2) | 0.35 |  |
|  | Pioglitazone vs Placebo | 0.28  (-0.18, 0.79) | 0.40  (-0.55, 1.3) | 0.30  (-0.09, 0.73) | 0.81 |  |
|  | Placebo vs Sitagliptin | -0.47  (-1.1, 0.13) | -0.26  (-2.7, 2.2) | -0.37  (-0.96, 0.15) | 0.86 |  |
| **HDL**  **HDL** | Ipragliflozin vs Pioglitazone | -0.44  (-6.7, 5.8) | 0.45  (-3.4, 4.) | 0.35  (-2.8, 3.0) | 0.80 |  |
|  | Ipragliflozin vs Placebo | -2.4  (-5.6, 0.82) | -3.4  (-9.8, 3.1) | -2.5  (-5.2, 0.02) | 0.78 |  |
|  | Liraglutide vs Metformin | 1.4  (-2.1, 4.7) | -5.5  (-10.0, -0.87) | -0.98  (-4.2, 1.8) | **0.01** |  |
|  | Liraglutide vs Pioglitazone | -8.0  (-16.0, 0.48) | 0.35  (-2.9, 3.9) | -0.76  (-4.0, 2.4) | 0.07 |  |
|  | Liraglutide vs Placebo | -5.0  (-9.2, -0.84) | -1.8  (-6.3, 2.3) | -3.5  (-6.6, -0.54) | 0.29 |  |
|  | Liraglutide vs Sitagliptin | -3.0  (-8.3, 2.2) | -3.6  ( -21., 13.) | -3.1  (-8.0, 1.8) | 0.94 |  |
|  | Metformin vs Pioglitazone | 2.7  (-1.7, 7.0) | -2.1  (-6.0, 1.9) | 0.24  (-2.6, 3.3) | 0.11 |  |
|  | Metformin vs Placebo | -0.60  (-4.9, 3.5) | -4.1  (-8.1, -0.03) | -2.5  (-5.3, 0.43) | 0.23 |  |
|  | Pioglitazone vs Placebo | -2.8  (-4.6, -0.80) | -2.7  (-6.9, 2.0) | -2.8  (-4.4, -0.97) | 0.96 |  |
|  | Placebo vs Sitagliptin | -0.4  (-17.0, 16.) | 0.44  (-5.4, 6.5) | 0.46  (-5.2, 6.0) | 0.92 |  |
| **LDL** | Ipragliflozin vs Pioglitazone | 7.7  ( -12.0, 27.0) | -13.0  (-26.0, -0.05) | -8.1  (-18.0, 7.2) | 0.07 |  |
|  | Ipragliflozin vs Placebo | -11.0  ( -23.0, 0.20) | 9.7  ( -11.0, 30.0) | -7.8  (-17.0, 6.9) | 0.07 |  |
|  | Liraglutide vs Metformin | -8.8  ( -21.0, 2.6) | 4.9  ( -14.0, 22.0) | -5.2  (-15.0, 5.1) | 0.19 |  |
|  | Liraglutide vs Pioglitazone | -0.28  ( -29.0, 29.0) | -6.1  (-18.0, 8.2) | -5.3  (-16.0, 7.3) | 0.72 |  |
|  | Liraglutide vs Placebo | 0.03  (-22.0, 22.0) | -6.3  (-19.0, 8.1) | -4.8  ( -16.0, 7.1) | 0.62 |  |
|  | Liraglutide vs Sitagliptin | 1.0  (-20.0, 22.0) | -14.  (-38.0, 12.0) | -4.8  (-21.0, 12.0) | 0.37 |  |
|  | Metformin vs Pioglitazone | -9.9  (-22.0, 3.2) | 9.9  (-2.7, 24.0) | -0.04  (-9.3, 10.0) | **0.03** |  |
|  | Metformin vs Placebo | 5.3  (-8.2, 19.0) | -4.4  (-18.0, 11.0) | 0.42  (-8.6, 11.0) | 0.31 |  |
|  | Pioglitazone vs Placebo | -0.41  (-9.3, 7.8) | 4.0  ( -13.0, 21.0) | 0.49  (-6.5, 7.0) | 0.61 |  |
|  | Placebo vs Sitagliptin | -6.9  (-29.0, 15.0) | 7.6  ( -18.0, 31.0) | -0.12  (-17.0, 16.0) | 0.39 |  |
| **Weight**  **Weight** | Ipragliflozin vs Pioglitazone | 3.8  (-4.6, 13.0) | 5.5  (1.5, 8.7) | 5.3  (1.8, 8.1) | 0.73 |  |
|  | Ipragliflozin vs Placebo | 2.0  (-1.2, 5.2) | 0.58  (-8.4, 9.6) | 2.0  (-0.84, 4.6) | 0.75 |  |
|  | Liraglutide vs Metformin | 1.9  (-1.8, 5.6) | 5.6  (0.89, 10.0) | 3.4  (0.41, 6.2) | 0.21 |  |
|  | Liraglutide vs Pioglitazone | 11.0  (5.6, 17.) | 6.8  (3.2, 9.7) | 7.7  (4.8, 10.) | 0.15 |  |
|  | Liraglutide vs Placebo | 4.3  (0.52, 7.7) | 4.4  (0.55, 8.9) | 4.4  (1.8, 6.9) | 0.95 |  |
|  | Liraglutide vs Sitagliptin | 1.9  (-4.2, 7.9) | 3.6  (-7.2, 15.) | 2.5  (-2.8, 7.7) | 0.79 |  |
|  | Metformin vs Pioglitazone | 1.9  (-2.0, 5.7) | 7.1  (3.0, 11.0) | 4.4  (1.4, 7.2) | 0.07 |  |
|  | Metformin vs Placebo | 3.6  (-3.8, 11.) | 0.47  (-2.7, 3.8) | 1.0  (-1.9, 4.1) | 0.44 |  |
|  | Pioglitazone vs Placebo | -3.6  (-5.6, -1.1) | -2.5  (-6.5, 1.8) | -3.4  (-5.1, -1.3) | 0.62 |  |
|  | Placebo vs Sitagliptin | -1.1  (-8.5, 6.4) | -2.4  (-10.0, 5.4) | -1.8  (-7.3, 3.5) | 0.82 |  |

**The reported results are displayed with effect size and 95% confidence interval (95% CI). Weighted mean difference (WMD) is applied to continuous results; BMI: body mass index; FPG: fasting plasma glucose; HbA1c: glycosylated hemoglobin; HDL: high density lipoprotein; LDL: low density lipoprotein; AST: alanine aminotransferase ; ALT: alanine aminotransferase.**

**Fig s2 The trajectory map, density map and convergent diagnostic diagram of AST**


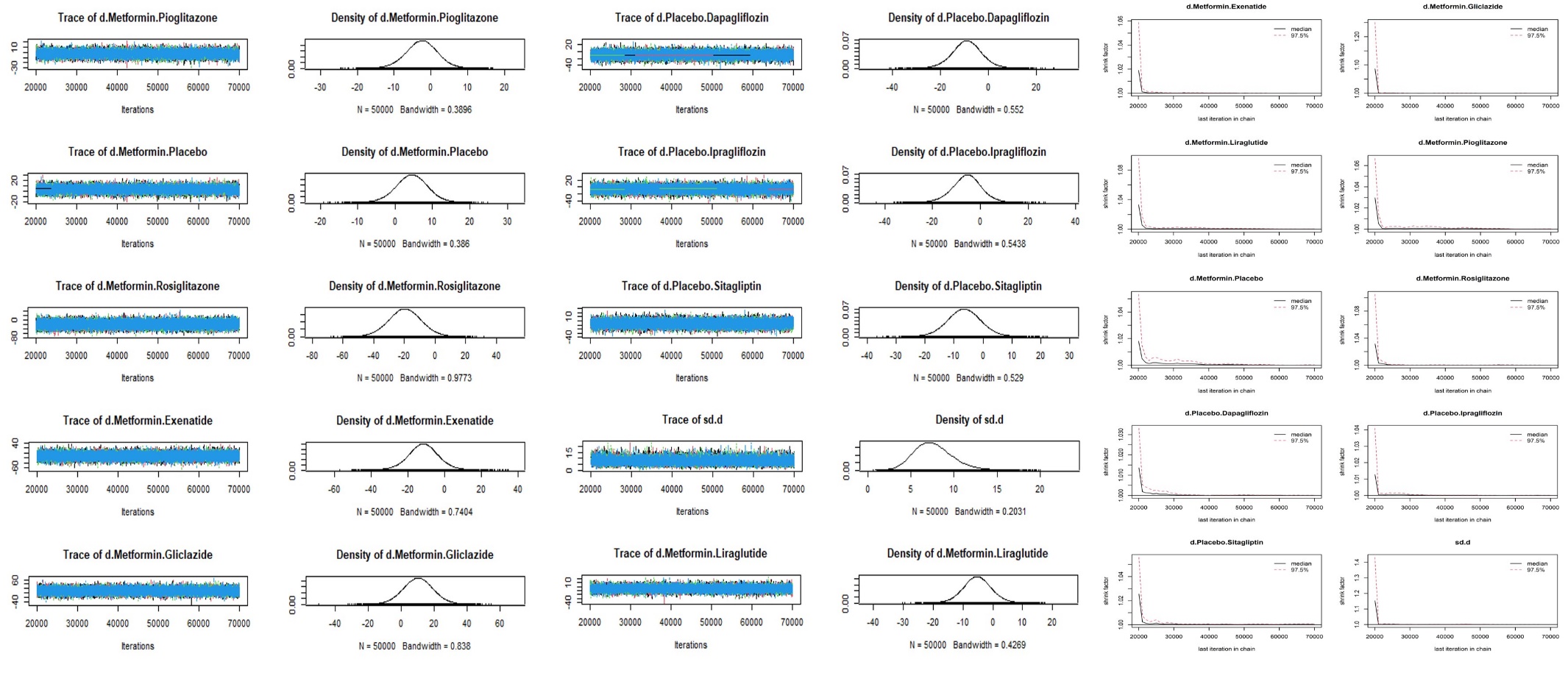


**Fig s2 The trace map, density map and convergent diagnostic diagram of AST**


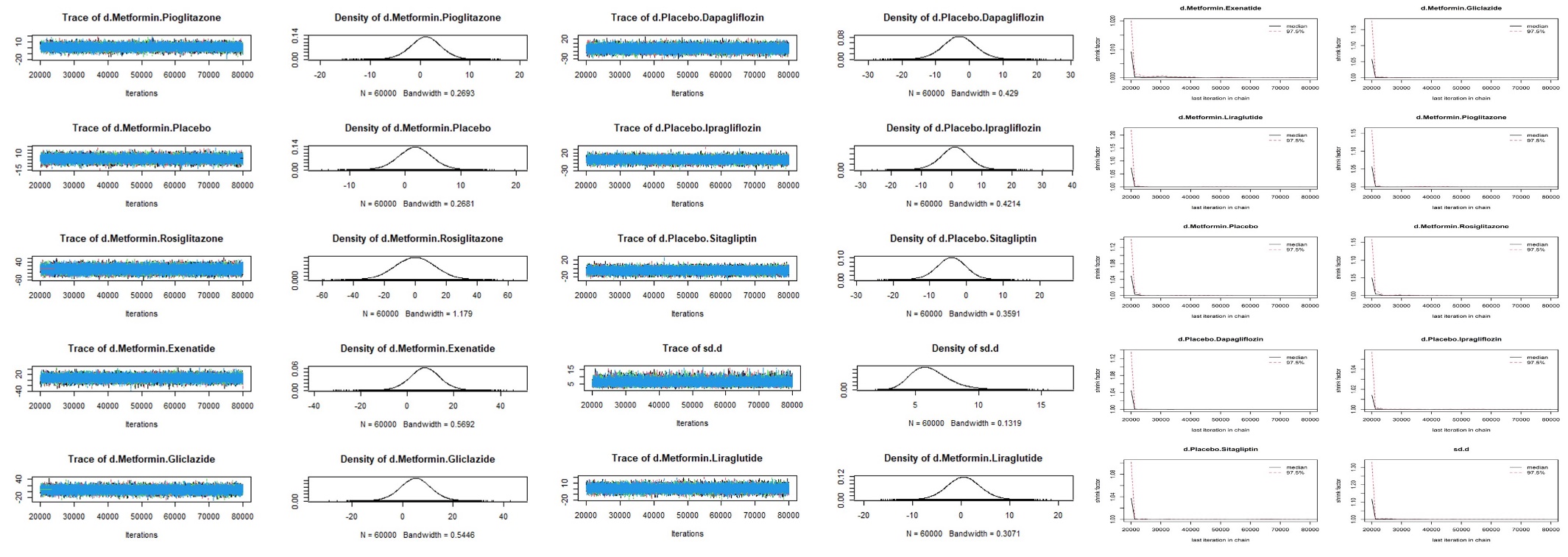


**Fig s3 The trace map, density map and convergent diagnostic diagram of Triglyceride**


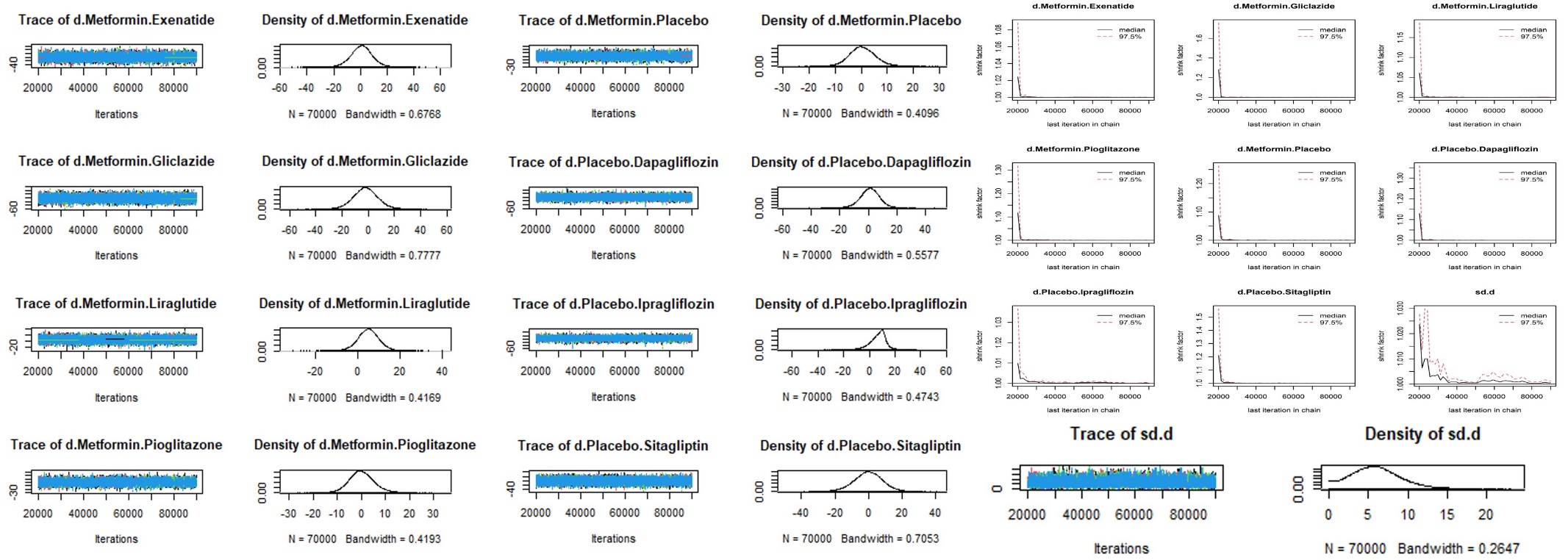


**Fig s4 The trace map, density map and convergent diagnostic diagram of BMI**


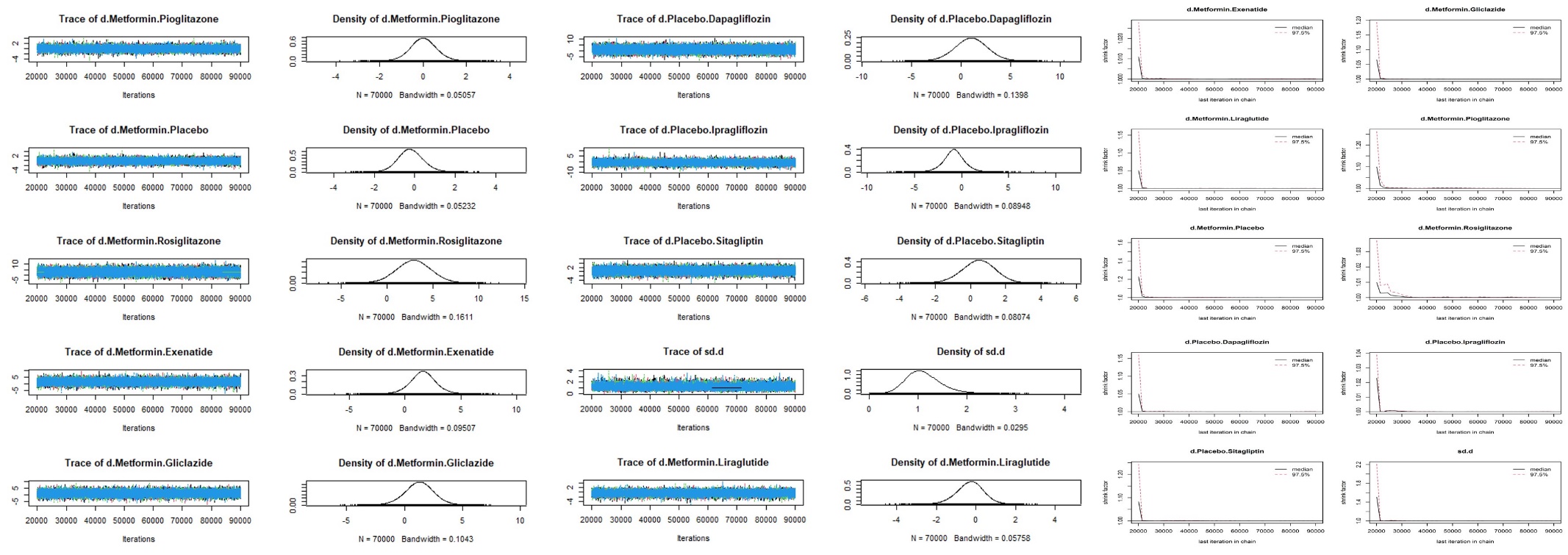


**Fig s5 The trace map, density map and convergent diagnostic diagram of FPG**


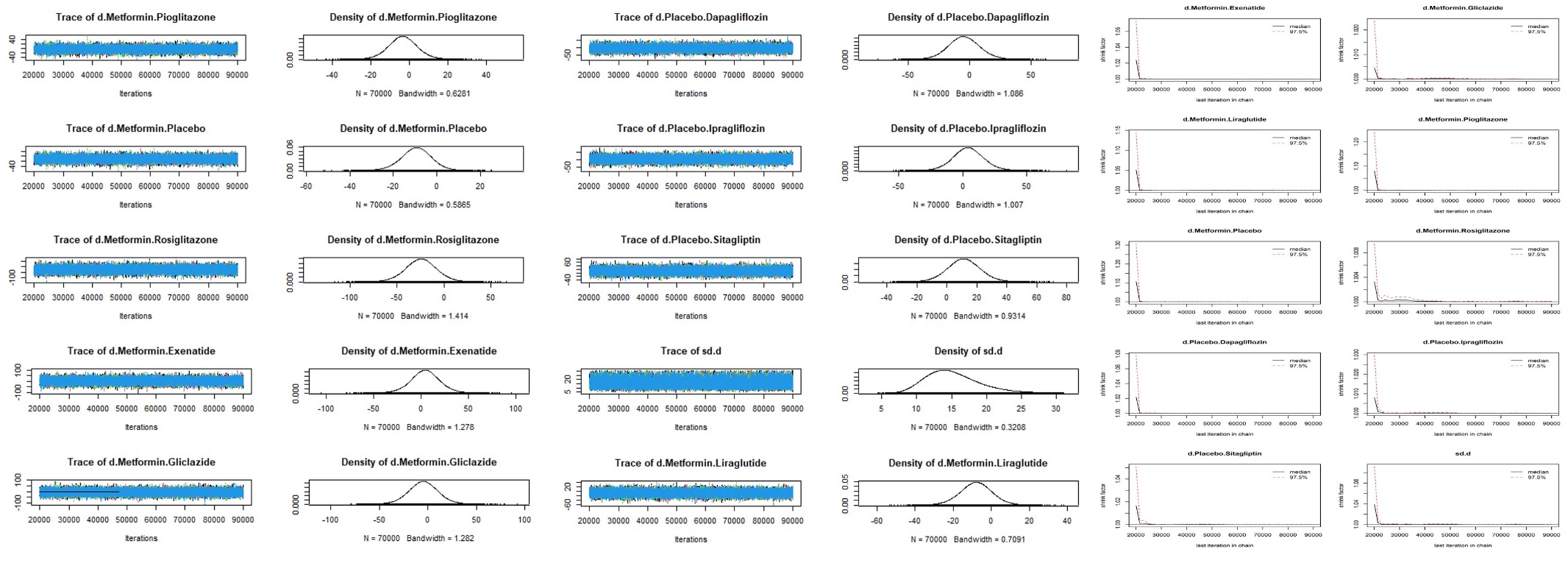


**Fig s6 The trace map, density map and convergent diagnostic diagram of HbA1c**


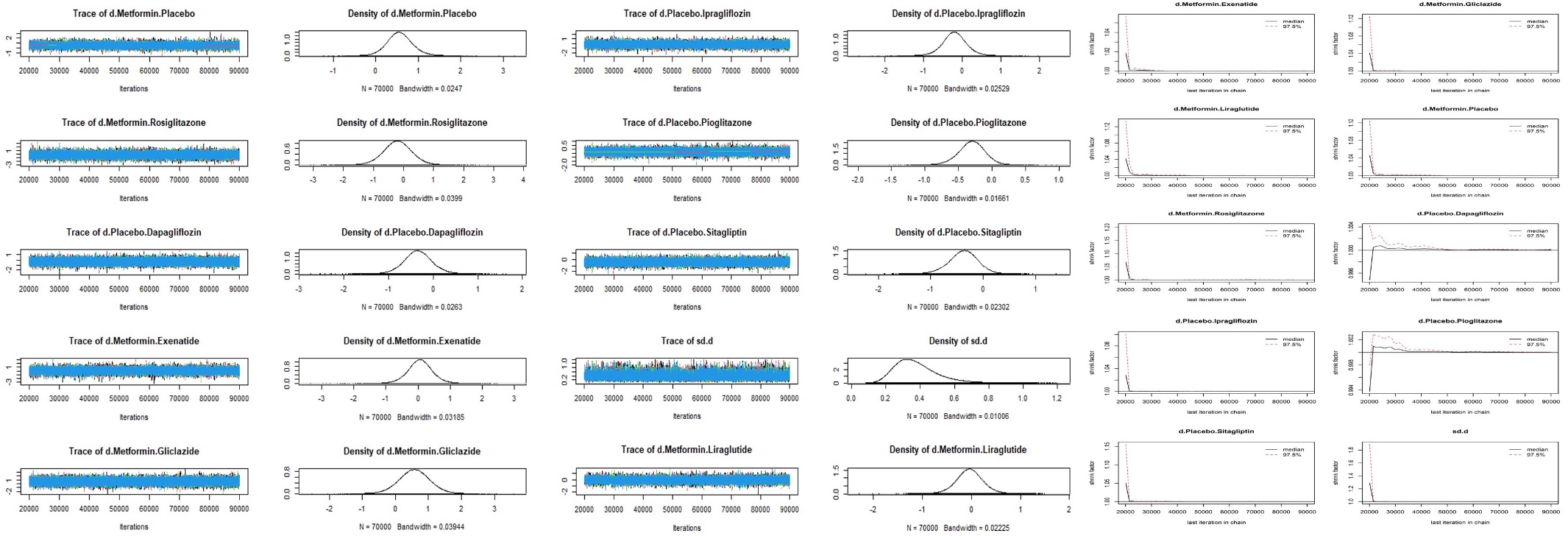


**Fig s7 The trace map, density map and convergent diagnostic diagram of HDL**


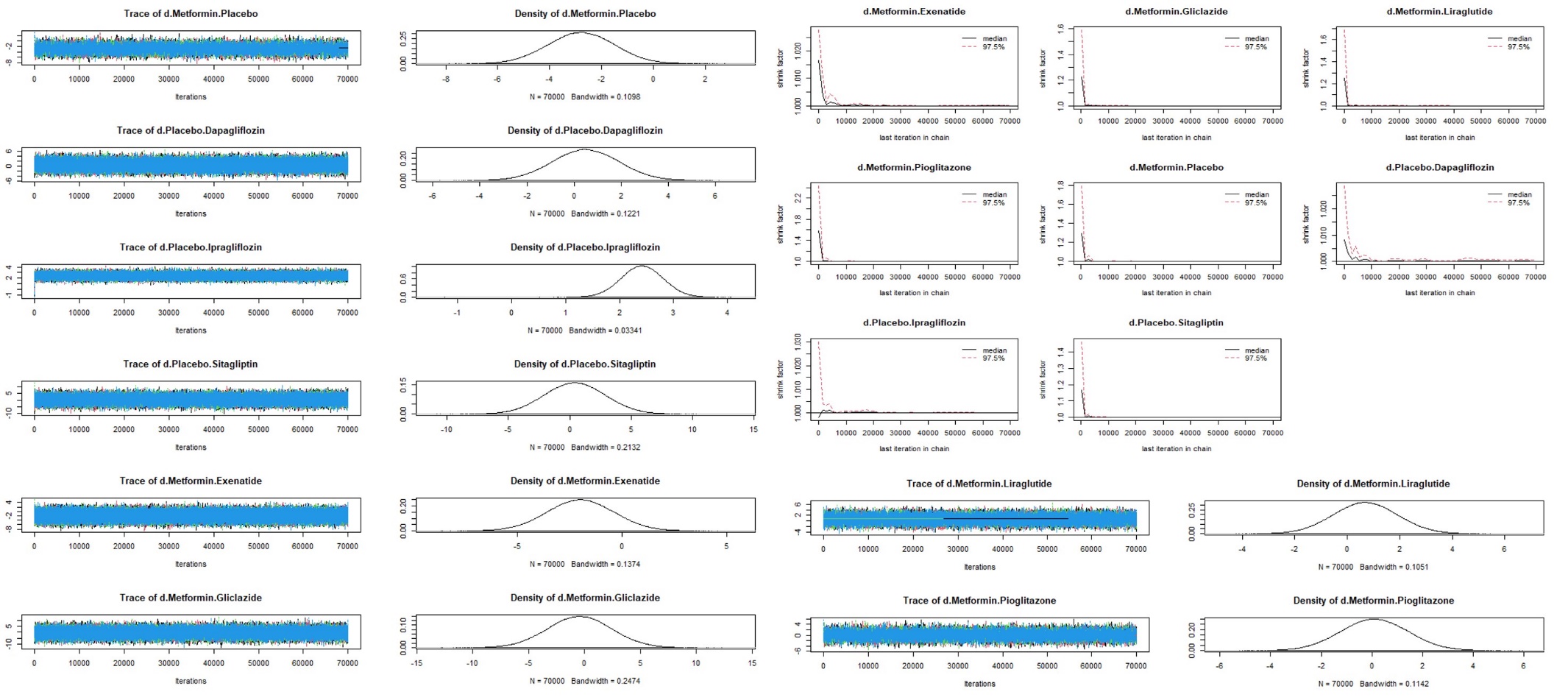


**Fig s8 The trajectory map, density map and convergent diagnostic diagram of LDL**


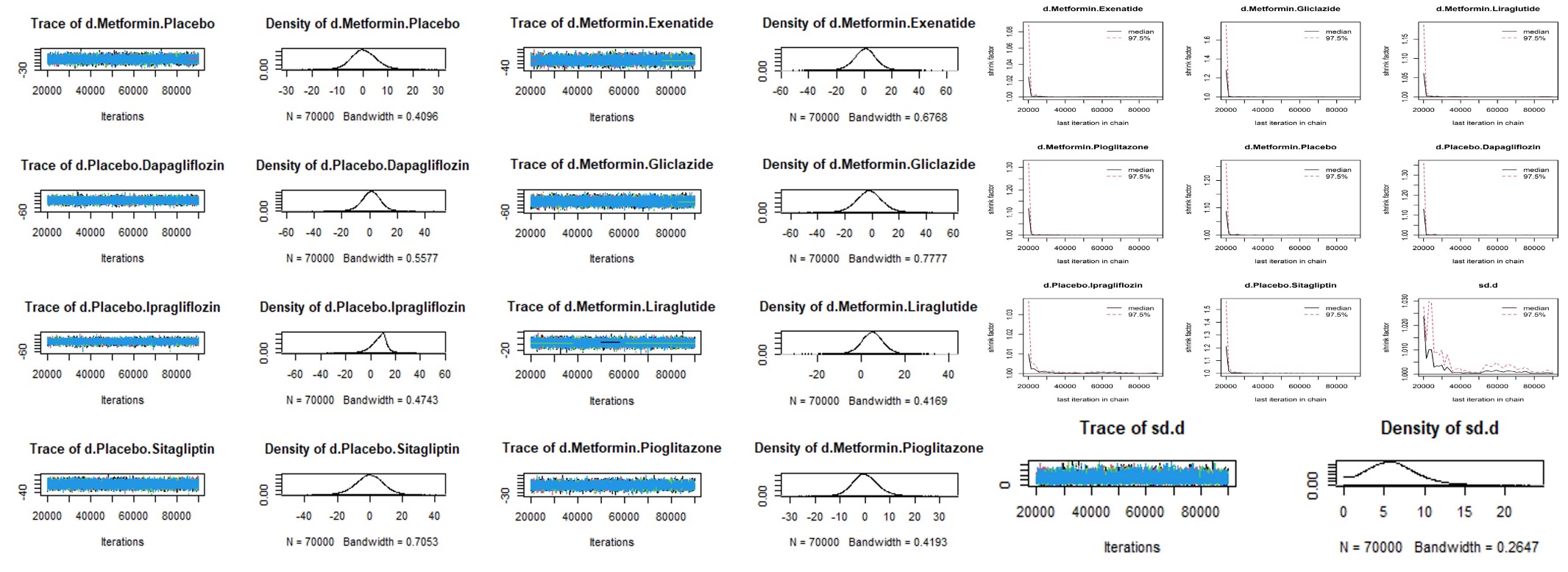


**Fig s9 The trajectory map, density map and convergent diagnostic diagram of Weight**


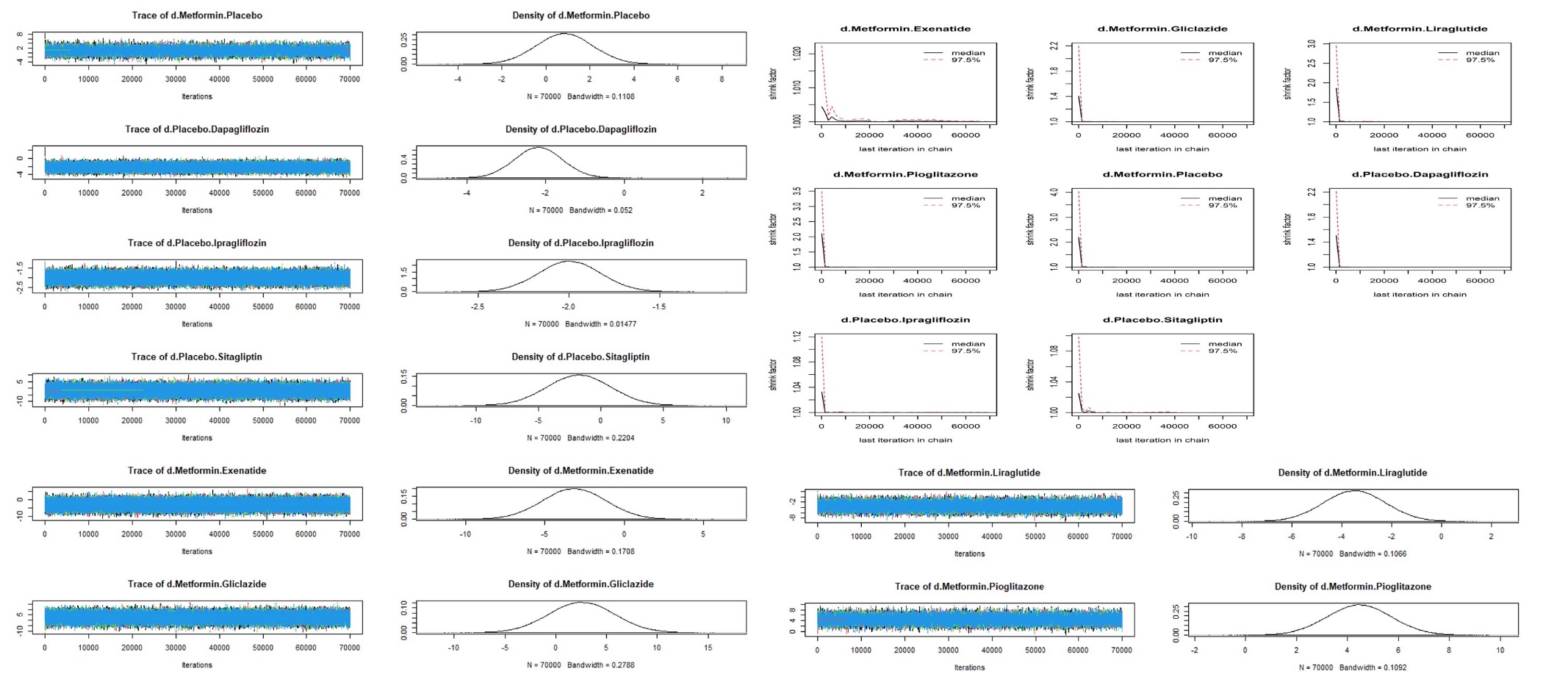

Supplement: Supplementary file 1 [file DataSheet_1.docx]
